# Effects of 31 FDA approved small-molecule kinase inhibitors on isolated rat liver mitochondria

**DOI:** 10.1007/s00204-016-1918-1

**Published:** 2016-12-28

**Authors:** Jun Zhang, Alec Salminen, Xi Yang, Yong Luo, Qiangen Wu, Matthew White, James Greenhaw, Lijun Ren, Matthew Bryant, William Salminen, Thomas Papoian, William Mattes, Qiang Shi

**Affiliations:** 1 Division of Systems Biology, National Center for Toxicological Research, Food and Drug Administration, 3900 NCTR Road, Jefferson, AR 72079 USA; 2 Biomedical Engineering 2016, University of Arkansas, Fayetteville, AR 72701 USA; 3 Division of Biochemical Toxicology, National Center for Toxicological Research, Food and Drug Administration, 3900 NCTR Road, Jefferson, AR 72079 USA; 4 ProNatural Brands LLC, 1174 Southwest 5th Avenue, Boca Raton, FL 33432 USA; 5 Division of Cardiovascular and Renal Products, Office of New Drugs I, Center for Drug Evaluation and Research, Food and Drug Administration, 10903 New Hampshire Avenue, Silver Spring, MD 20993 USA

**Keywords:** Hepatotoxicity, Kinase inhibitor, Drug induced liver injury, Mitochondrion, Submitochondrial particles

## Abstract

The FDA has approved 31 small-molecule kinase inhibitors (KIs) for human use as of November 2016, with six having black box warnings for hepatotoxicity (BBW-H) in product labeling. The precise mechanisms and risk factors for KI-induced hepatotoxicity are poorly understood. Here, the 31 KIs were tested in isolated rat liver mitochondria, an in vitro system recently proposed to be a useful tool to predict drug-induced hepatotoxicity in humans. The KIs were incubated with mitochondria or submitochondrial particles at concentrations ranging from therapeutic maximal blood concentrations (Cmax) levels to 100-fold Cmax levels. Ten endpoints were measured, including oxygen consumption rate, inner membrane potential, cytochrome c release, swelling, reactive oxygen species, and individual respiratory chain complex (I–V) activities. Of the 31 KIs examined only three including sorafenib, regorafenib and pazopanib, all of which are hepatotoxic, caused significant mitochondrial toxicity at concentrations equal to the Cmax, indicating that mitochondrial toxicity likely contributes to the pathogenesis of hepatotoxicity associated with these KIs. At concentrations equal to 100-fold Cmax, 18 KIs were found to be toxic to mitochondria, and among six KIs with BBW-H, mitochondrial injury was induced by regorafenib, lapatinib, idelalisib, and pazopanib, but not ponatinib, or sunitinib. Mitochondrial liability at 100-fold Cmax had a positive predictive power (PPV) of 72% and negative predictive power (NPV) of 33% in predicting human KI hepatotoxicity as defined by product labeling, with the sensitivity and specificity being 62% and 44%, respectively. Similar predictive power was obtained using the criterion of Cmax ≥1.1 µM or daily dose ≥100 mg. Mitochondrial liability at 1–2.5-fold Cmax showed a 100% PPV and specificity, though the NPV and sensitivity were 32% and 14%, respectively. These data provide novel mechanistic insights into KI hepatotoxicity and indicate that mitochondrial toxicity at therapeutic levels can help identify hepatotoxic KIs.

**Electronic supplementary material:**

The online version of this article (doi:10.1007/s00204-016-1918-1) contains supplementary material, which is available to authorized users.

## Introduction

Drug-induced liver injury (DILI) is a major safety concern for patients, clinicians, pharmaceutical companies, and regulatory agencies. Nearly 50% of orally administered drugs on the market have been linked to DILI, though the causality is not always clear (Weng et al. 2015b). DILI is of particular importance for small-molecule kinase inhibitors (KIs), a group of recently developed drugs used to treat various types of cancer and other diseases such as idiopathic pulmonary fibrosis. They may be recommended for long-term usage or to be used until unacceptable toxicity such as DILI is observed. In the product labeling, almost 20% (6 out of 31) of FDA approved KIs have a black box warning, the strongest safety warnings issued by the FDA, due to DILI, and 71% (22 out of 31) have a “Warnings and Precautions” section for DILI. The mechanisms and risk factors for KI hepatotoxicity may include metabolic activation (Castellino et al. 2012; Teo et al. 2015), direct mitochondrial damage (Weng et al. 2015a), oxidative stress (Xue et al. 2012), inhibition of hepatic transporters (Feng et al. 2009), and genetic variations in drug metabolism enzymes (Sugiyama et al. 2015; Takimoto et al. 2013). However, previous studies only investigated a small number (less than 10 in total) of KIs, and the results from different groups cannot be compared directly, as different models and platforms were used. A comprehensive study involving all FDA approved KIs is needed to help better understand KI hepatotoxicity regarding its mechanism and prediction for DILI.

Mitochondrial damage has long been recognized as an important mechanism for DILI (Meyers et al. 1988). Recent studies have gone a step further and provided evidence that mitochondrial liability was predictive of a chemical’s potential to induce DILI in humans (Aleo et al. 2014; Porceddu et al. 2012). However, few KIs have been examined regarding mitochondrial toxicity. In the present study, all 31 FDA approved KIs were tested in isolated rat liver mitochondria at concentrations normalized to blood levels at or above therapeutic doses in humans. Mitochondrial functions that were measured in the current study included: oxygen consumption rate, inner membrane potential (MMP), cytochrome c release which reflects outer membrane integrity, swelling, reactive oxygen species (ROS), and the five individual respiratory chain complex (RCC I–V) activities. The results suggest that direct mitochondrial toxicity may contribute to the mechanism of hepatotoxicity induced by some KIs, but the predictive power of KI-induced mitotoxicity for clinical hepatotoxicity is rather limited.

## Materials and methods

### Chemicals and reagents

KIs were obtained from three vendors including Medkoo Biosciences (Chapel Hill, NC), Selleck Chemicals (Houston, TX), and LC laboratories (Woburn, MA). Rhodamine 123 was from Cayman Chemical (Ann Arbor, MI). Cytochrome c profiling ELISA kit was from purchased Abcam Inc (Cambridge, MA). The ROS probe 5-(and-6)-chloromethyl-2′,7′-dichlorodihydrofluorescein diacetate, acetyl ester (CM-H2DCFDA) was purchased from Thermo Fisher Scientific Inc (Grand Island, NY). Dimethyl sulfoxide (DMSO), sodium dodecyl sulfate (SDS), and other chemicals were obtained from Sigma-Aldrich (St Louis, MO).

### Characterization of commercial KIs by HPLC-Mass spectrometry

KI stock solutions were prepared in DMSO and further diluted to 1 μg/ml as working standards with acetonitrile containing 0.1% formic acid. The solutions were stored at −20 °C until analysis. Ten μl of each KI solution was injected into a Waters e2695 Alliance HPLC System coupled with both a Waters 2998 Photodiode Array (PDA) Detector and an ACQUITY QDa Mass Detector. Each analyte was eluted on a Waters Atlantis T3 C18 column (4.6 × 150 mm, 5 μm) at 40 °C using an isocratic mobile phase composed of 20% 10 mM ammonium formate and 80% acetonitrile both containing 0.1% formic acid at a flow rate of 0.5 ml/min. The eluate was monitored for up to 10 min by the PDA and by mass spectrometry with the electrospray ion source operating in the positive ion mode (ESI+) using MS scans of 100–1000 Da.

### Animal care

Sprague–Dawley rats (220–350 g) were obtained from the U.S. Food and Drug Administration National Center for Toxicological Research (NCTR) breeding colony. Animal care and experimental procedures were approved by the NCTR Institutional Animal Care and Use Committee in accordance with the National Institutes of Health (NIH) “Guide for the Care and Use of Laboratory Animals” (http://www.ncbi.nlm.nih.gov/books/NBK54050). Preliminary experiments showed that mitochondria from male and female rats responded similarly to KI treatment. Therefore, only male rats were used in subsequent studies.

### Isolation of rat liver mitochondria

Rat liver mitochondria were prepared as described previously (Weng et al. 2015a). Rats were anesthetized by an intraperitoneal injection of Nembutal and the liver perfused with PBS to remove the blood. The liver was then cut into small pieces and homogenized using a Dounce type homogenizer in the ice-cold isolation buffer (IB) containing 200 mM sucrose, 10 mM Tris-MOPS, 1 mM EGTA (pH 7.4). The sample was then centrifuged at 600*g* for 10 min. The supernatant was further centrifuged at 7500*g* for 10 min, and the pellets was washed once with the IB and centrifuged again at 7500*g* for 10 min to collect mitochondria. The protein concentration was measured using the Bradford method (Kruger 1994). Before drug treatment, the protein concentration was adjusted to 1 mg/ml using appropriate buffer. The purity of mitochondria was ascertained by detecting four mitochondrion-specific proteins and the cytosolic protein glyceraldehyde-3-phosphate dehydrogenase (GAPDH) using Western blot (Gusdon et al. 2015). Briefly, mitochondrial and cytosolic fractions from four different preparations using four rats were diluted using the Laemmli protein loading buffer and subject to SDS-PAGE with 4–20% gradient gels. The proteins were blotted to PVDF membranes and detected using antibodies against GAPDH, ATP5A, UQCRC2, SDHB, and NDUFB8.

### Drug treatment

The KI stock solutions were prepared in DMSO and aliquoted in 0.5 ml tubes before storing at −20 °C. The aliquoted stock solutions were kept for less than one month and each tube was used only once. The KIs were added to the mitochondria at 1:1000 dilutions. The final concentrations for the majority of KIs were 100, 50, 30, 20, 10, 5, 2.5, and 1-fold of Cmax as reported in the product labeling and drug approval packages at the official FDA website (https://www.accessdata.fda.gov/scripts/cder/drugsatfda). The Cmax data were from clinical trials which used FDA recommend dosages, route of administration, and duration. Several KIs were tested at lower concentrations because of the low solubility in DMSO or the tendency to precipitate when added to test buffers or the interference with the assays. The final concentration of DMSO for drug treatment was always 0.1%, and control treatments were 0.1% DMSO.

### Mitochondrial oxygen consumption

A published procedure was followed to measure mitochondrial oxygen consumption (Weng et al. 2015a). Briefly, mitochondria were incubated with the KIs for 5 min, and then split evenly and loaded to the sample tubes of two Oxytherm systems (Hansatech Instruments Ltd), with one system used for measuring glutamate/malate driven respiration and the other succinate-driven respiration, both of which were defined as state 4 respiration. After 3 min, ADP was added to measure the maximal oxygen consumption rate, which was defined as state 3 respiration. The buffer used for these experiments contained 125 mM KCl, 10 mM Tris-MOPS, 1 mM KH_2_PO_4_, 10 µM EGTA-Tris, pH7.4, and was referred to as respiration buffer (RB). The oxygen consumption rate of DMSO-treated samples (controls) was set as 1.

### Measurement of MMP

MMP was measured using Rhodamine 123 as described in a previous report (Buron et al. 2010). Briefly, mitochondria were suspended in RB supplemented with 5 mM succinic acid, 2 µM rotenone and 10 µM Rhodamine 123, and then KIs were added. The fluorescence (excitation 485 nm, emission 535 nm) was recorded every 1 min for 30 min using a Synergy 2 Multi-Mode microplate reader form BioTek (Winooski, VT).

### Mitochondrial ROS production

ROS was measured using the fluorescence probe CM-H2DCFDA (Mattiasson 2004). Briefly, freshly isolated mitochondria were diluted to 1 mg/ml using IB buffer containing 4 µM CM-H2DCFDA. After 30 min, the samples were centrifuged at 7500 *g* for 10 min and the resulting pellet re-suspended in a buffer containing 150 mM KCl, 5 mM KH_2_PO_4_, 5 mM Tris, 10 µM CaCl_2_, 2 µM rotenone, and 5 mM succinate (pH 7.4). KIs were then added, and 100 µM CaCl_2_ was used as a positive control. The fluorescence (excitation 490 nm, emission 530 nm) was measured every 1 min for 30 min by using a Synergy 2 Multi-Mode microplate reader form BioTek (Winooski, VT). The signal from DMSO-treated samples was set as 1.

### Activities of RCC I–V

The activities of RCC I–V were determined using previously published procedures (Kirby et al. 2007; Weng et al. 2014). Submitochondrial particles were prepared by subjecting the intact mitochondria to three successive freeze–thaw cycles (Kirby et al. 2007). KIs were incubated with submitochondrial particles (1 mg/ml) for 15 min and then RCC I–V activities measured. The final KI concentrations ranged from 1–100 fold Cmax. The activities of DMSO-treated samples (controls) were set as 1. RCC I activity was measured using a buffer containing 25 mM potassium phosphate, 5 mM MgCl_2_, pH7.2, 0.25% bovine serum albumin (BSA), 0.13 mM β-Nicotinamide adenine dinucleotide, reduced dipotassium salt (NADH), 2 mM potassium cyanide (KCN), 2 µg/ml antimycin A and 65 µM ubiquinone 1, which was supplemented with or without 2 µg/ml rotenone. The difference between the activity from rotenone-containing buffer and rotenone-absent buffer was considered as RCC I activity. RCC II activity was measured in a buffer containing 25 mM potassium phosphate, 5 mM MgCl_2_, pH7.2, 20 mM sodium succinate, 50 µM 2,6-dichloroindophenol sodium salt hydrate, 2 mM KCN, 2 µg/ml antimycin A, 2 µg/ml rotenone, and 65 µM ubiquinone 1. RCC III activity was determined with a buffer containing 25 mM potassium phosphate, pH 7.2, 1 mM n-dodecyl-β-D-maltoside, 1 mM KCN, 1 µg/ml rotenone, 100 µM reduced-decylubiquinone, 15 µM oxidized cytochrome c, and 0.1% BSA. RCC IV activity was measured using a buffer containing 25 mM potassium phosphate, pH 7.2, 1 mM n-dodecyl-β-D-maltoside, 15 µM reduced cytochrome c. RCC V was measured with a buffer containing 40 mM Tris, 10 mM EGTA, pH 8.0, 0.2 mM NADH, 2.5 mM phospho(enol)pyruvic acid monopotassium salt, 2.5 µg/ml antimycin A, 5 mM MgCl_2_, 5 U/ml lactate dehydrogenase, 5 U/ml pyruvate kinase, and 2.5 mM ATP, which was supplemented with or without 2 µg/ml oligomycin A. The difference between the activity from oligomycin A-containing buffer and oligomycin A-absent buffer was considered as RCC V activity. For measuring RCC I/V and II activity, the changes in absorbance at 340 and 600 nm, respectively, were determined every 1 min for 6 min. For measuring RCC III and RCC IV, the changes in absorbance at 550 nm were measured every 10 s for 2 min.

### Mitochondrial swelling

A previously published procedure was followed in detail for measuring mitochondrial swelling (Weng et al. 2015a).

### Cytochrome *c* release assay

Freshly isolated liver mitochondria (1 mg/ml protein) were re-suspended in RB supplemented with 5 mM succinic acid and 2 µM rotenone, and then KIs were added, with DMSO used as a vehicle control and alamethicin as a positive control whose signal was set as 100 (Buron et al. 2010). The samples were incubated for 30 min with gentle shaking. After centrifugation 10,000*g* for 10 min, the supernatant and pellets were collected and kept at −80 °C. The cytochrome c level in the supernatant was determined using the cytochrome c profiling ELISA Kit from Abcam Inc. (Cambridge, MA) following the manufacturer’s manual. As the antibody only reacts with denatured cytochrome c, it is of critical importance to incubate the supernatant with a buffer containing 1% SDS prior to measurement.

### Data analysis

The means and standard deviations were obtained from at least three batches of mitochondria isolated from three different Sprague–Dawley male rats. The difference among treatment groups were analyzed by one-way or two-way ANOVA followed by Dunnett’s test using the software GraphPad Prism 6 (San Diego, California). A *p* value less than 0.05 was considered statistically meaningful. The positive prediction value (PPV), negative prediction value (NPV), sensitivity, and specificity were calculated as previously described (Porceddu et al. 2012).

## Results

### Overview of tested KIs

All FDA approved small-molecule KIs were retrieved from the FDA official website at http://www.fda.gov/Drugs/DevelopmentApprovalProcess/HowDrugsareDevelopedandApproved/DrugandBiologicApprovalReports/NDAandBLAApprovalReports/ucm373420.htm. As of November 2016, the FDA has approved 31 KIs for human use. The Cmax, recommended daily dose, date of approval, and DILI potential of these drugs are summarized in Table 1. The recommended daily dose of these KIs ranged from 2 to 1375 mg, and the therapeutic Cmax ranged from 0.04 to 132.80 µM, a span of about three orders of magnitude, highlighting the importance of normalizing the test concentrations to Cmax for in vitro studies comparing KI toxicities. The chemical structure and molecular weight of KIs tested in this study were ascertained using mass spectrometry-based technology (Supplementary Fig. 1). No quality issues were identified with these commercial KIs.

**Table 1:** 31 KIs tested in the present study

Drug name	Approval date as a new molecular entity	Cmax (µM)	Recommended daily dose (mg/day)	DILI potential (based on labeling)	labeling for DILI
Imatinib	10-May-01	2.00	600	1	W&P: fatal DILI
Gefitinib	5-May-03	0.27	250	1	W&P: fatal DILI
Erlotinib	18-Nov-04	3.44	150	1	W&P: fatal DILI
Sorafenib	20-Dec-05	4.30	400	1	W&P: fatal DILI
**Sunitinib**	26-Jan-06	0.12	44	1	BBW: fatal DILI
Dasatinib	28-Jun-06	0.18	120	0	NA
**Lapatinib**	13-Mar-07	4.18	1375	1	BBW: fatal DILI
Nilotinib	29-Oct-07	4.27	700	1	W&P: ALT↑/monitoring
**Pazopanib**	19-Oct-09	132.80	800	1	BBW: fatal DILI
Vandetanib	6-Apr-11	1.80	300	0	NA
Vemurafenib	17-Aug-11	0.13	960	1	W&P: hepatic impairment/ALT monitoring
Crizotinib	26-Aug-11	0.91	500	1	W&P: fatal DILI
Ruxolitinib	16-Nov-11	1.20	25	0	NA
Axitinib	27-Jan-12	0.07	10	1	W&P: ALT↑/monitoring
Bosutinib	4-Sep-12	0.38	500	1	W&P: ALT↑/monitoring
**Regorafenib**	27-Sep-12	8.08	160	1	BBW: fatal DILI
Tofacitinib	6-Nov-12	0.09	10	1	W&P: ALT↑/monitoring
Cabozantinib	29-Nov-12	2.58	100	0	NA
**Ponatinib**	14-Dec-12	0.14	45	1	BBW: fatal DILI
Trametinib	29-May-13	0.04	2	0	NA
Dabrafenib	29-May-13	2.84	300	0	NA
Afatinib	12-Jul-13	0.04	40	1	W&P: ALT↑/monitoring
Ibrutinib	12-Feb-14	0.37	490	0	NA
Ceritinib	29-Apr-14	1.81	750	1	W&P: ALT↑/monitoring
**Idelalisib**	23-Jul-14	4.64	300	1	BBW: fatal DILI
Nintedanib	15-Oct-14	0.06	300	1	W&P: ALT↑/monitoring
Palbociclib	3-Feb-15	0.24	125	0	NA
Lenvatinib	13-Feb-15	0.68	24	1	W&P: fatal DILI
Cobimetinib	10-Nov-15	0.51	60	1	W&P: ALT↑/monitoring
Osimertinib	13-Nov-15	0.50	80	0	NA
Alectinib	11-Dec-15	1.37	1200	1	W&P: ALT↑/monitoring

*W&P* warnings and precautions, *BBW* black box warnings, *ALT* alanine aminotransferase, *NA* not available. The 31 drugs are all FDA approved KIs for human use. Definition of DILI potential: 1, BBW and/or W&P indicated that the drug cased DILI; 0, no DILI information available in the labeling. The six KIs with a BBW for hepatotoxicity are highlighted in red

The known pharmacological targets of these KIs were collected from http://www.brimr.org/PKI/PKIs.htm and drug labeling from https://dailymed.nlm.nih.gov/dailymed/index.cfm (Supplementary Table 1). The pharmacological targets associated with at least two approved KIs were listed in Table 2. The targets were listed according to the percentage of DILI positive KIs among each group. An interesting observation is that all 3 ALK inhibitors and 91% (10 out of 11) of PDGFR inhibitors are hepatotoxic. It is likely that KIs targeting these two pathways have exceptionally high risks of liver injury. As for several other targets, such as EphR, Sec and Tie2, only 50% of the corresponding KIs are hepatotoxic. This is in line with the observation that about 50% (478 out of 975) of oral drugs have been associated with DILI (Weng et al. 2015b), indicating that targeting these kinase pathways is unlikely to cause additional DILI risks. However, definitive conclusions cannot be drawn at this time, as the number of KIs in each group is relatively small.

**Table 2:** Pharmacological targets and DILI potentials of FDA approved KIs

Targets	DILI positive KIs	DILI negative KIs	% of DILI positive KIs (# of positive/negative KIs)
ALK	Crizotinib; ceritinib; alectinib	None	100% (3/0)
PDGFR	Imatinib; sorafenib; sunitinib*; nilotinib; pazopanib*; axitinib; regorafenib*; ponatinib*; nintedanib; lenvatinib	Dasatinib	91% (10/1)
BCR-Abl	Imatinib; nilotinib; bosutinib; regorafenib*; ponatinib*	Dasatinib	83% (5/1)
VEGFR	Sorafenib; sunitinib*; pazopanib*; axitinib; regorafenib*; ponatinib*; nintedanib; lenvatinib	Vandetanib; cabozantinib	80% (8/2)
Flt3	Sorafenib; sunitinib*; ponatinib*; nintedanib	Cabozantinib	80% (4/1)
Kit	Imatinib; sorafenib; sunitinib*; Pazopanib*; regorafenib*; ponatinib *; lenvatinib	Dasatinib; cabozantinib	78% (7/2)
RET	Sorafenib; sunitinib*; regorafenib*; ponatinib*; lenvatinib; alectinib	Vandetanib; cabozantinib	75% (6/2)
B-Raf	Sorafenib; vemurafenib; regorafenib*	Dabrafenib	75% (3/1)
EGFR	Gefitinib; erlotinib; lapatinib*; afatinib	Vandetanib; osimertinib	67% (4/2)
EphR	Regorafenib*; ponatinib*	Dasatinib; vandetanib	50% (2/2)
Src	Bosutinib; ponatinib*	Dasatinib; vandetanib	50% (2/2)
Tie2	Regorafenib*; ponatinib*	Vandetanib; cabozantinib	50% (2/2)
MEK	Cobimetinib	Trametinib	50% (1/1)
JAK	Tofacitinib	Ruxolitinib	50% (1/1)
Lck	Pazopanib	Dasatinib	50% (1/1)

* indicates the KIs with a black box warning for hepatotoxicity

### KI effects on mitochondrial oxygen consumption

Before drug treatment, the purity of mitochondria from selective rats was examined using Western blot. As shown in Supplementary Fig. 2A, the cytosolic protein GAPDH was only detectable in cytosolic fractions but not mitochondrial preparations. In contrast, the four mitochondrion-specific proteins including ATP5A, UQCRC2, SDHB and NDUFB8 were only detectable in mitochondrial preparations but not cytosolic fractions (Supplementary Fig. 2B). These results indicate that our mitochondria were devoid of cytosolic contamination.

**Fig. 1:**
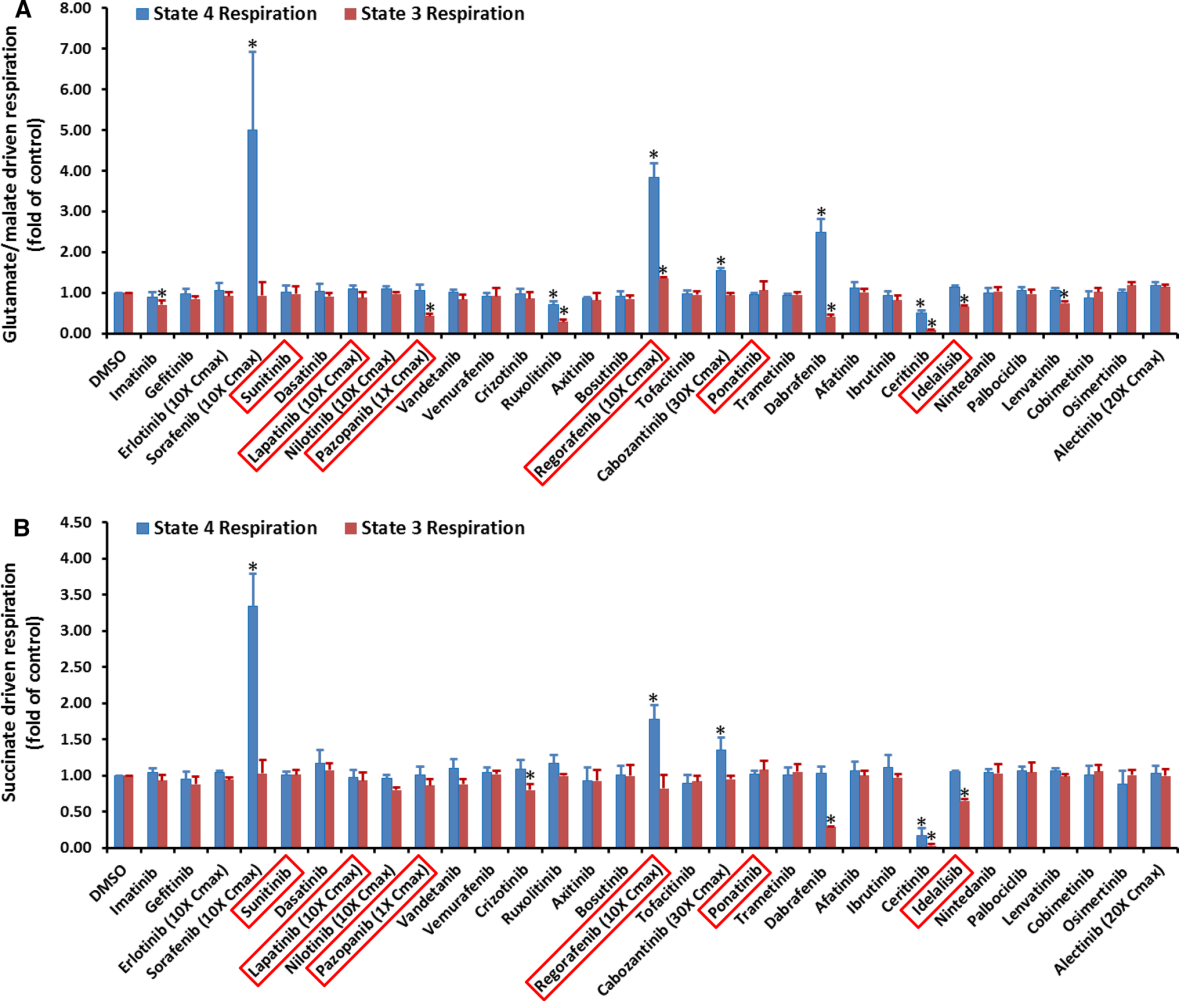
KI effects on mitochondrial oxygen consumption. Rat liver mitochondria were incubated with KIs at 100-fold Cmax or the maximal testable concentrations as indicated. The KIs in the *X*-axis were listed according to their approval date. The six KIs with a black box warning for hepatotoxicity were highlighted in a *red box*. The *Y*-axis represents the fold changes of oxygen consumption after KI treatment as compared to DMSO-treated samples. **a**, **b** Represent the oxygen consumption rate measured using glutamate/malate and succinate, respectively. Data are means and standard derivations from three separate experiments. *p < 0.05 as compared to DMSO-treated samples (color figure online)

Oxygen consumption is one of the most important functions of mitochondria that can be affected by hepatotoxic drugs. Figure 1 shows the mitochondrial oxygen consumption rate after KI treatment at the highest tested concentrations, and the dose dependent effects of 11 KIs that showed significant effects in Fig. 1 are presented in Table 3. The remaining data are included in Supplementary Table 1. Three KIs, including sorafenib, pazopanib and regorafenib, significantly affected oxygen consumption starting at concentrations equal to Cmax, while two KIs, dabrafenib and cabozantinib, began to show detrimental effects staring from tenfold Cmax (Table 3). Ruxolitinib and three other KIs (imatinib, ceritinib and lenvatinib) disrupted oxygen consumption starting from 20- and 30-fold Cmax, respectively. Idelalisib was non-effective until 50-fold Cmax, and crizotinib only affected oxygen consumption at 100-fold Cmax (Table 3).

**Table 3:** Dose response of KI effects on mitochondrial oxygen consumption

Drugs (fold of Cmax)	Glutamate/malate driven respiration		Succinate-driven respiration	
	State 4	State 3	State 4	State 3
Sorafenib				
10	5.00 ± 1.92*	0.93 ± 0.34	3.34 ± 0.45*	1.03 ± 0.19
5	3.69 ± 0.12*	1.34 ± 0.04*	1.03 ± 0.08	0.40 ± 0.01*
2.5	4.92 ± 0.09*	1.57 ± 0.06*	1.43 ± 0.06*	0.62 ± 0.05*
1	2.55 ± 0.27*	1.15 ± 0.09	1.50 ± 0.21*	1.00 ± 0.10
Regorafenib				
10	3.84 ± 0.34*	1.36 ± 0.03*	1.78 ± 0.19*	0.82 ± 0.19
5	3.82 ± 0.72*	1.33 ± 0.04*	1.69 ± 0.09*	0.83 ± 0.03
2.5	5.51 ± 0.35*	2.51 ± 0.04*	3.59 ± 0.14*	0.84 ± 0.04
1	7.45 ± 0.17*	2.33 ± 0.06*	2.95 ± 0.08*	1.05 ± 0.07
Pazopanib				
1	1.05 ± 0.15	0.43 ± 0.06*	1.01 ± 0.11	0.86 ± 0.09
Dabrafenib				
100	2.49 ± 0.32*	0.41 ± 0.05*	1.03 ± 0.09	0.28 ± 0.01*
50	2.42 ± 0.21*	0.56 ± 0.05*	1.69 ± 0.30*	0.44 ± 0.10*
30	2.22 ± 0.24*	0.71 ± 0.03*	1.52 ± 0.12*	0.59 ± 0.07*
20	1.59 ± 0.05*	0.90 ± 0.05	1.31 ± 0.02*	0.65 ± 0.01*
10	1.35 ± 0.19*	0.94 ± 0.04	1.38 ± 0.13*	0.97 ± 0.07
Cabozantinib				
30	1.55 ± 0.06*	0.94 ± 0.05	1.35 ± 0.18*	0.95 ± 0.05
20	1.30 ± 0.15*	0.92 ± 0.05	1.24 ± 0.05*	0.94 ± 0.06
10	1.46 ± 0.08*	1.01 ± 0.03	1.23 ± 0.11*	1.03 ± 0.01
Ruxolitinib				
100	0.71 ± 0.08*	0.28 ± 0.06*	1.17 ± 0.12	0.99 ± 0.03
50	0.97 ± 0.20	0.49 ± 0.06*	1.07 ± 0.07	0.87 ± 0.08
30	1.00 ± 0.09	0.54 ± 0.02*	1.08 ± 0.09	1.05 ± 0.11
20	0.81 ± 0.05	0.62 ± 0.05*	1.00 ± 0.14	0.90 ± 0.13
Ceritinib				
100	0.50 ± 0.06*	0.08 ± 0.02*	0.17 ± 0.10*	0.03 ± 0.02*
50	1.21 ± 0.12*	0.55 ± 0.03*	0.77 ± 0.07*	0.45 ± 0.08*
30	1.12 ± 0.13	0.88 ± 0.05	0.70 ± 0.12*	0.41 ± 0.18*
Imatinib				
100	0.90 ± 0.12	0.70 ± 0.11*	1.04 ± 0.06	0.93 ± 0.07
50	0.90 ± 0.06	0.67 ± 0.03*	0.99 ± 0.06	0.90 ± 0.01
30	0.93 ± 0.04	0.71 ± 0.02*	0.98 ± 0.02	0.94 ± 0.04
Lenvatinib				
100	1.06 ± 0.06	0.74 ± 0.04*	1.07 ± 0.03	0.99 ± 0.03
50	0.93 ± 0.01	0.70 ± 0.02*	0.96 ± 0.03	0.90 ± 0.01
30	0.95 ± 0.06	0.76 ± 0.05*	0.94 ± 0.01	0.86 ± 0.04
Idelalisib				
100	1.13 ± 0.05	0.66 ± 0.03*	1.05 ± 0.01	0.65 ± 0.03*
50	1.00 ± 0.08	0.76 ± 0.04*	0.97 ± 0.04	0.83 ± 0.06
Crizotinib				
100	0.97 ± 0.14	0.86 ± 0.15	1.09 ± 0.12	0.80 ± 0.08*

The signal from DMSO-treated samples was set as 1. * *p* < 0.05 as compared to DMSO-treated samples. The next lower concentrations showed no effects. KIs not listed also showed no effects

At the highest tested concentrations, four drugs, including sorafenib, regorafenib, cabozantinib and dabrafenib caused significant increases of state 4 respiration (Fig. 1), indicating these drugs uncoupled oxidative phosphorylation (OXPHOS). Seven KIs decreased state 3 respiration driven by glutamate/malate (Fig. 1a), and four KIs suppressed state 3 respiration driven by succinate (Fig. 1b), indicating these drugs were likely inhibitors of OXPHOS. Of note, ceritinib caused almost 100% inhibition of mitochondrial oxygen consumption, albeit at 100-fold Cmax.

### KI effects on activities of RCC I–V

**Fig. 2:**
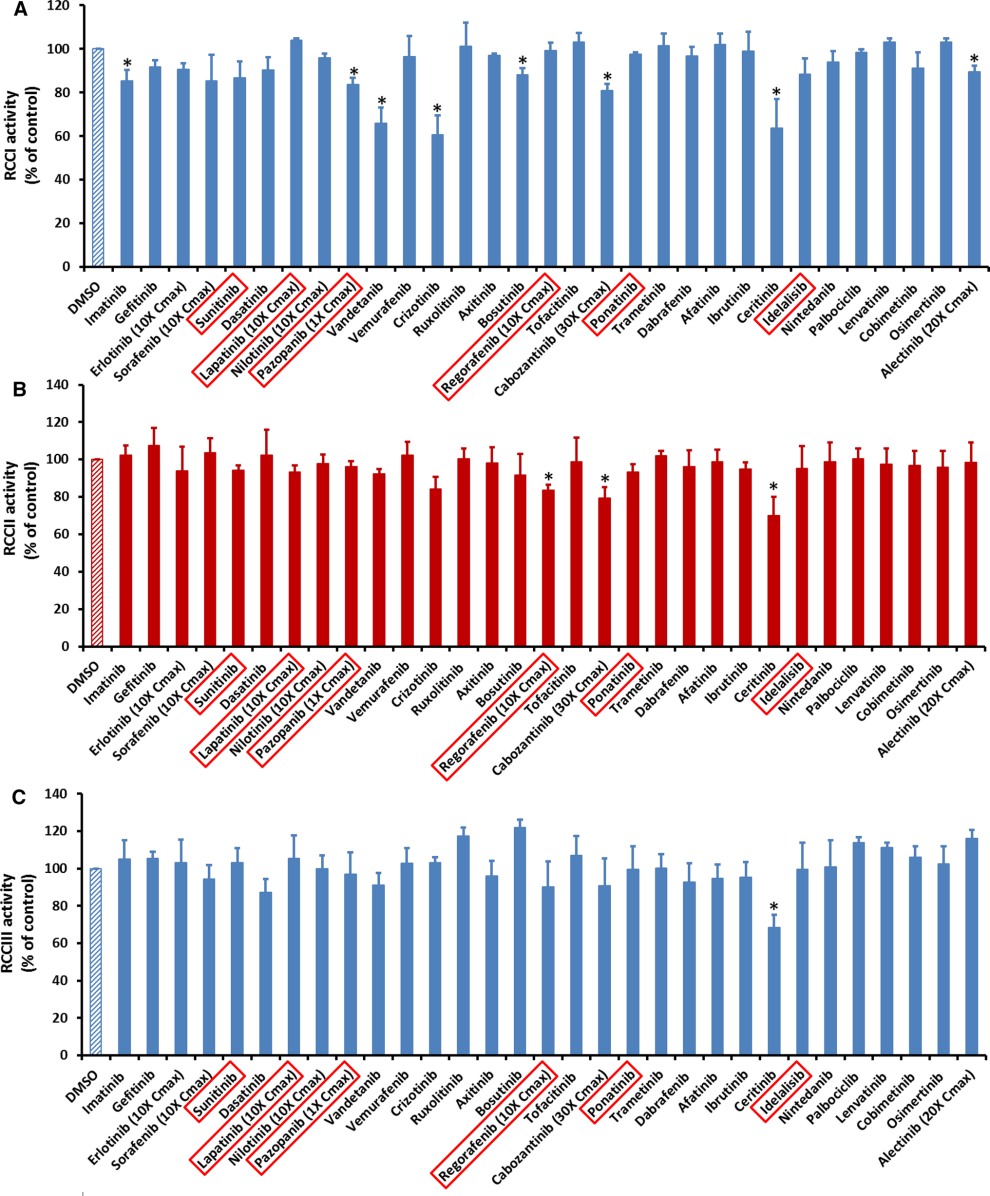

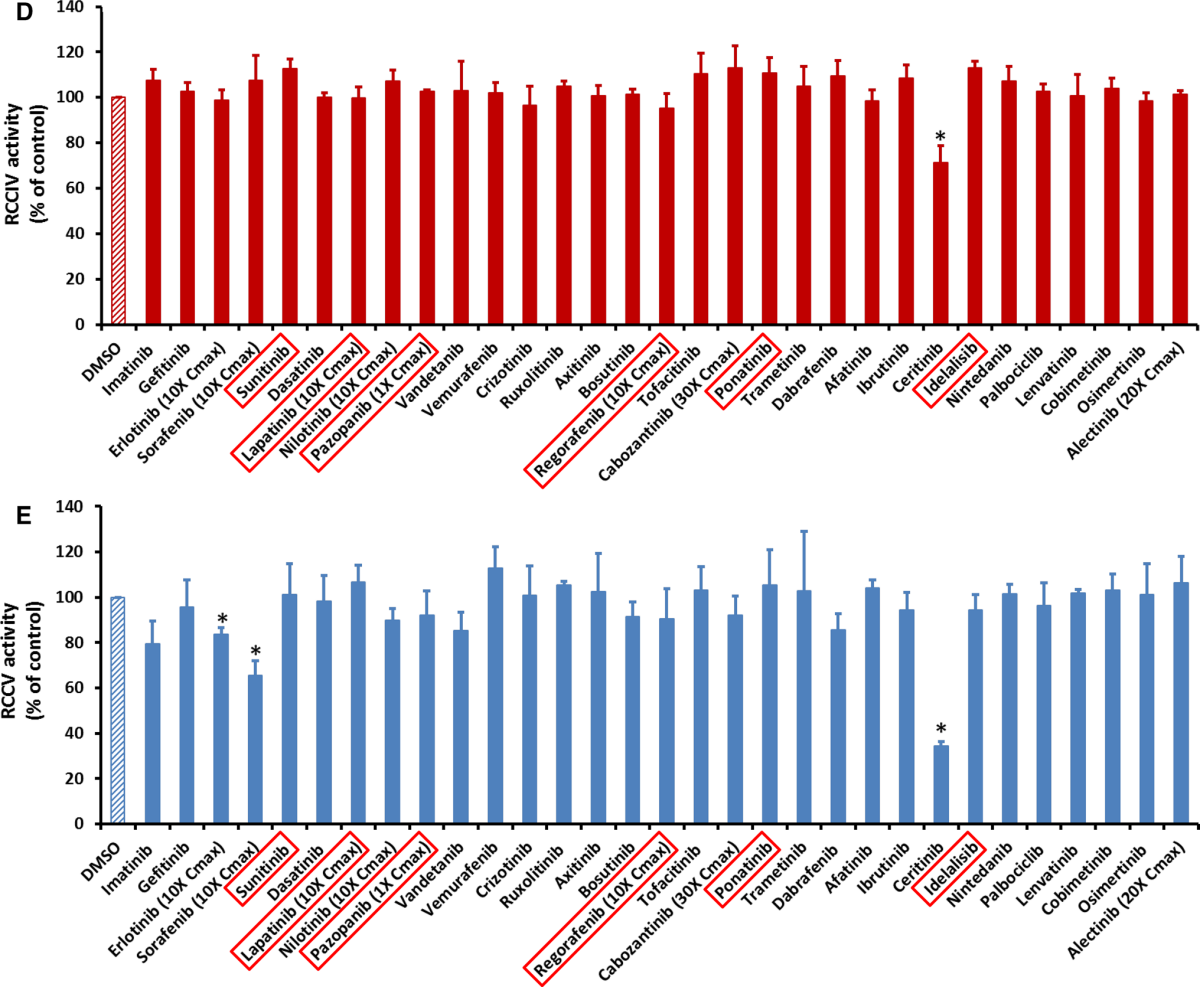
KI effects on activities of RCC I–V. Submitochondrial particles were incubated with KIs at 100-fold Cmax or the highest testable concentrations as. After 15 min, the activities of RCC I–V were measured. **a**, **b**, **c**, **d**, **e** Represents the activity of RCC I, II, III, IV, and V, respectively. The drugs in the *X*-axis were listed according to their approval date. The six KIs with a black box warning for hepatotoxicity were highlighted in a *red box*. The *Y*-axis represents the percentage of RCC activity after KI treatment as compared to DMSO-treated samples which were set as 100. Data are means and standard derivations from three independent experiments. *p < 0.05 as compared to DMSO-treated samples (color figure online)

The inhibitory effects of KIs on mitochondrial oxygen consumption might be due to the direct inhibition of RCC activities by KIs. To identify the specific RCCs inhibited by KIs, the activities of RCC I–V were measured using submitochondrial particles. KIs concentrations tested were the same as described for intact mitochondria. The results of all 31 KIs at the highest tested concentrations are shown in Fig. 2a–e, and the dose dependent responses for the 11 KIs that significantly inhibited RCCs were presented in Table 4. The “negative” results are included in Supplementary Table 1. Pazopanib was the only KI that inhibited RCCs at concentrations equal to Cmax, and sorafenib was the next toxic KIs as it started to inhibit RCCs at fivefold Cmax. Regorafenib and erlotinib began to suppress RCCs at 10- fold Cmax, and the other KIs showed detrimental effect only at concentrations equal or larger than 20-fold Cmax. At the highest tested concentrations, the number of KIs inhibiting RCC I, II, III, IV, and V was 8, 3, 1, 1, and 3, respectively. It was evident that RCC I was the major target for KI inhibition. The drug ceritinib was the only KI that caused inhibition of all five RCCs, and cabozantinib inhibited both RCC I and RCC II. For other KIs with RCC inhibition, the effects appeared protein complex-specific. For example, imatinib, regorafenib, and sorafenib only inhibited RCC I, RCC II and RCC V, respectively, but not other RCCs.

**Table 4:** Dose response of KI effects on activities of RCC I–V

Drugs (fold of Cmax)	RCC activity (100% of control)				
	I	II	III	IV	V
Pazopanib					
1	83.77 ± 2.98*	96.12 ± 2.96	96.90 ± 11.69	102.68 ± 0.57	92.22 ± 10.47
Sorafenib					
10	85.45 ± 11.93	103.68 ± 7.88	94.31 ± 7.47	107.65 ± 10.77	65.55 ± 6.34*
5	87.23 ± 10.61	101.40 ± 6.29	97.60 ± 5.50	101.55 ± 3.61	78.30 ± 4.68*
Erlotinib					
10	90.62 ± 2.87	93.97 ± 12.92	103.11 ± 12.25	98.77 ± 4.53	83.52 ± 2.96*
Regorafenib					
10	99.37 ± 3.35	83.50 ± 2.91*	90.31 ± 13.61	95.37 ± 6.45	90.36 ± 13.39
Crizotinib					
100	60.51 ± 8.90*	84.10 ± 6.53	103.28 ± 2.85	96.60 ± 8.40	100.80 ± 13.09
50	71.46 ± 4.28*	90.31 ± 4.38	98.02 ± 7.16	103.77 ± 5.67	91.95 ± 5.63
30	76.48 ± 2.18*	95.03 ± 1.23	106.26 ± 5.79	96.65 ± 4.14	98.65 ± 2.98
20	86.52 ± 2.70*	100.85 ± 3.65	99.67 ± 8.65	94.03 ± 9.67	95.77 ± 7.64
Alectinib					
20	89.39 ± 2.98*	98.44 ± 10.74	116.05 ± 4.72	101.46 ± 1.66	106.30 ± 11.74
Cabozantinib					
30	80.97 ± 2.99*	79.26 ± 6.01*	90.73 ± 14.60	113.10 ± 9.48	91.99 ± 8.51
Ceritinib					
100	63.74 ± 13.12*	69.91 ± 10.00*	68.48 ± 6.59*	71.46 ± 7.39*	34.25 ± 2.09*
50	94.04 ± 3.32	89.14 ± 6.26	103.80 ± 5.19	82.33 ± 3.68*	71.23 ± 9.65*
Vandetanib					
100	65.88 ± 7.15*	92.39 ± 2.47	91.23 ± 6.54	103.06 ± 12.96	85.15 ± 8.36
50	87.71 ± 0.58*	90.03 ± 5.23	99.20 ± 5.61	100.56 ± 2.67	95.65 ± 4.66
Imatinib					
100	85.30 ± 5.13*	102.36 ± 5.33	104.96 ± 10.18	107.65 ± 4.65	79.34 ± 10.06
Bosutinib					
100	88.03 ± 3.01*	91.74 ± 11.35	101.81 ± 4.43	101.25 ± 2.26	91.53 ± 6.49

The signal from DMSO-treated samples was set as 100. * *p* < 0.05 as compared to DMSO-treated samples. The next lower concentrations showed no effects. KIs not listed also showed no effects

### KI effects on MMP

**Fig. 3:**
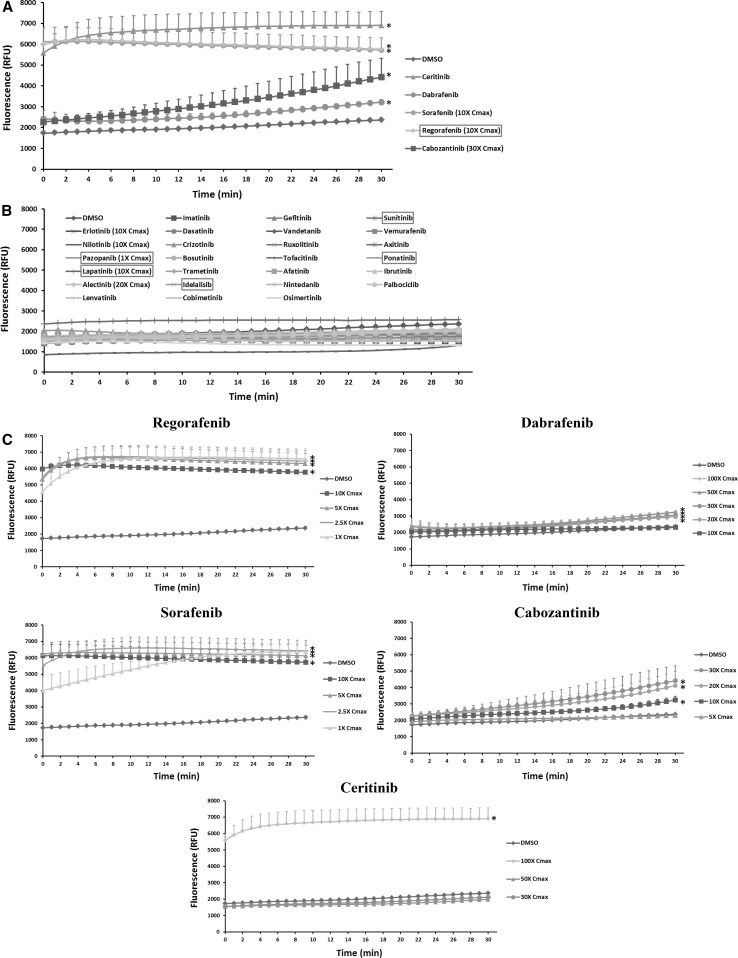
KI effects on mitochondrial inner membrane potential (MMP). Rat liver mitochondria were supplemented with 10 µM Rhodamine 123 and incubated with KIs at 100-fold Cmax or the highest testable concentrations as indicated. The fluorescence (excitation 485 nm, emission 535 nm) was recorded every 1 min for 30 min and presented in the *Y*-axis (**a**, **b**). **c** Shows the dose response of five KIs. In **a**, **c**, data are means and standard derivations from three separate experiments. *p < 0.05 as compared to DMSO-treated samples at the end of experiments. In **b**, data are means of two independent experiments

Uncoupling of mitochondrial respiration can disrupt MMP. Among 31 KIs tested, only five drugs including regorafenib, ceritinib, dabrafenib, sorafenib and cabozantinib caused a decrease in MMP at the highest concentrations tested (Fig. 3a), and the remaining drugs showed no effects at any of the concentrations tested (Fig. 3b; Supplementary Table 1). Among the five effective drugs, ceritinib showed the strongest potential. Dose–response studies showed that sorafenib and regorafenib started to decrease MMP at onefold Cmax, and cabozantinib and dabrafenib at tenfold and 20-fold Cmax, respectively, while ceritinib showed no effects at concentrations lower than 100-fold Cmax (Fig. 3c).

### KI effects on mitochondrial swelling

**Fig. 4:**
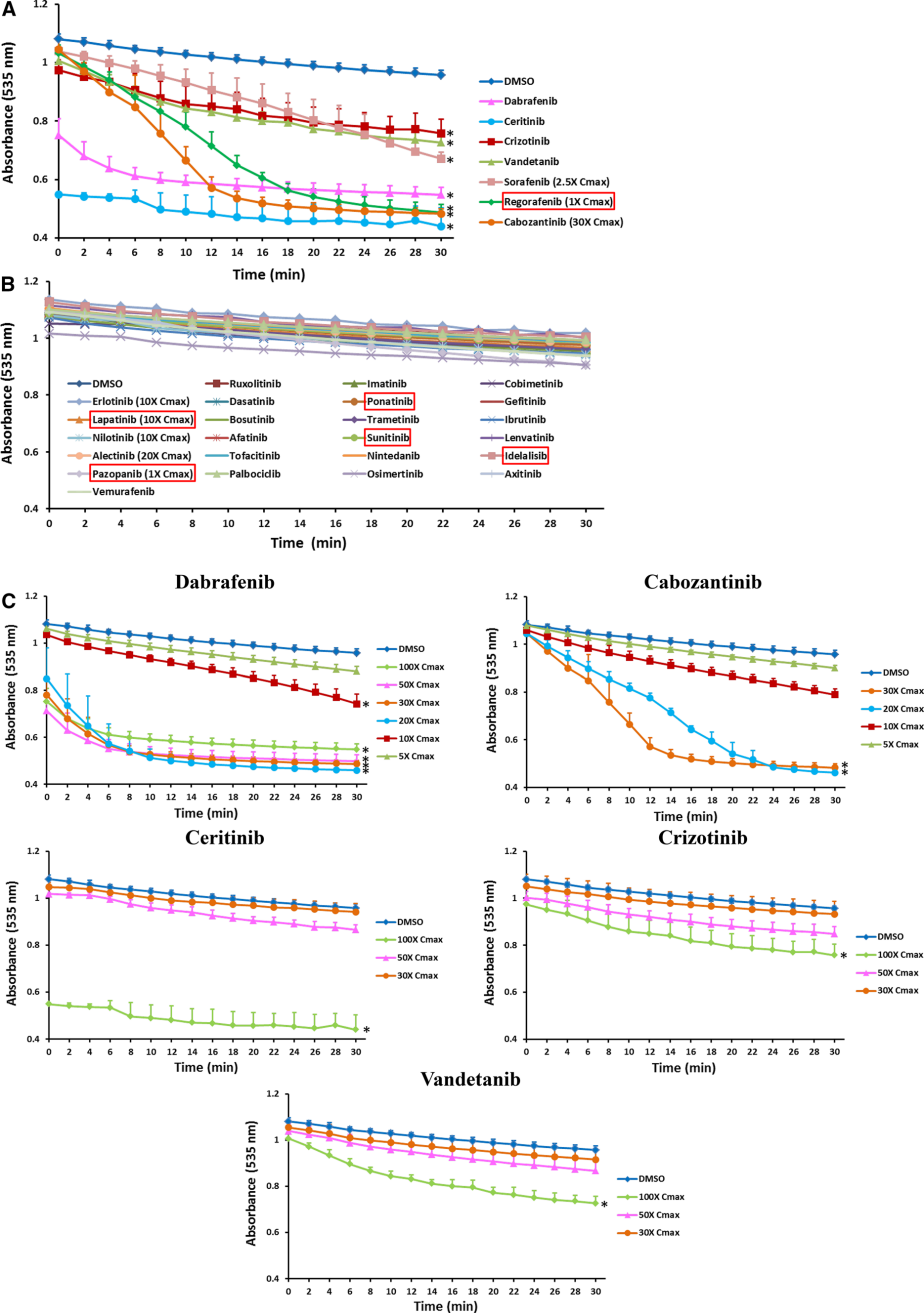
KI effects on mitochondrial swelling. Rat liver mitochondria were supplemented with 25 µM calcium chloride and incubated with KIs at 100-fold Cmax or the highest testable concentrations as indicated. The six KIs with a black box warning for hepatotoxicity were highlighted in a *red box*. Mitochondrial swelling was determined by monitoring the decrease in absorbance at 535 nm every 2 min for 30 min. **c** Shows the dose response of five KIs. **a**, **c** Data are means and standard derivations of three separate experiments. *p < 0.05 as compared to DMSO-treated samples at the end of experiments. **b** Data are means of two independent experiments (color figure online)

Altered permeability of the mitochondrial inner membrane can lead to swelling. Figure 4a shows that seven KIs including regorafenib, sorafenib, dabrafenib, cabozantinib, ceritinib, crizotinib and vandetanib caused significant mitochondrial swelling at the highest concentrations tested. Other drugs tested showed no effects on mitochondrial swelling at any of the concentrations tested (Fig. 4b; Supplementary Table 1). The dose response of five effective KIs is presented in Fig. 4c. Only one drug regorafenib caused mitochondrial swelling at onefold Cmax, and sorafenib caused swelling at 2.5-fold Cmax (Supplementary Table 1). Of note, though regorafenib at onefold Cmax caused almost maximal swelling, it was not possible for us to test it reliably at the next higher concentrations (2.5-fold Cmax), because absorbance at 535 nm of the drug itself was observed. This was also the case for sorafenib. Therefore, the dose response curves were not presented in the main text. Figure 4c shows that dabrafenib and cabozantinib started to cause swelling at tenfold and 20-fold Cmax, respectively. The remaining three drugs including ceritinib, crizotinib, and vandetanib only caused swelling at 100-fold Cmax.

### KI effects on cytochrome c release

**Fig. 5:**
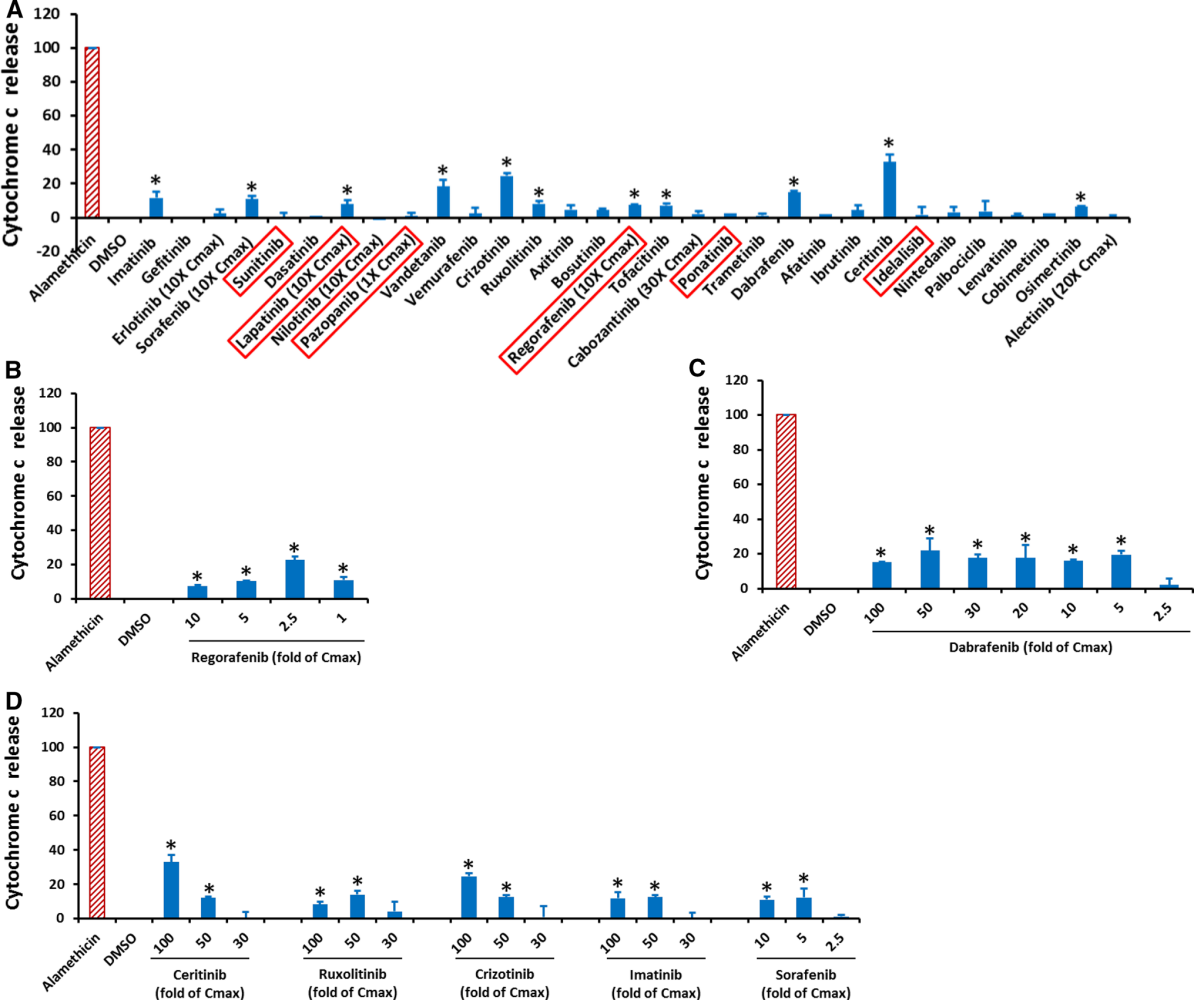
KI effects on cytochrome c release. Rat liver mitochondria were incubated with KIs at 100-fold Cmax or the highest testable concentrations as indicated. Alamethicin was used to induce complete cytochrome c release, and its signal was set as 100. After 30 min, the mitochondria were pelleted and cytochrome c level in the supernatant was determined using an ELISA Kit. The drugs in the *X*-axis were listed according to their approval date. The six KIs with a black box warning for hepatotoxicity were highlighted in a* red box* (**a**). **b**–**d** Are the dose response of seven KIs. Data are means and standard derivations from three independent experiments. *p < 0.05 as compared to DMSO-treated samples (color figure online)

Changes in the permeability of mitochondrial outer membrane can lead to cytochrome c release. Figure 5a shows that 11 KIs induced cytochrome c release at the highest concentrations tested, and the remaining 20 KIs were ineffective. The dose response of seven KIs was shown in Fig. 5b–d. Only regorafenib started to trigger cytochrome c release at onefold Cmax (Fig. 5b), and two drugs, sorafenib and dabrafenib, caused cytochrome c release starting at fivefold Cmax (Fig. 5c, d). Lapatinib caused cytochrome release only at tenfold Cmax, though higher concentrations cannot be tested due to its limited solubility (Supplementary Table 1). Four KIs including ceritinib, ruxolitinib, crizotinib and imatinib began to cause cytochrome c at 50-fold Cmax (Fig. 5d), and three KIs including vandetanib, tofacitinib, osimertinib only showed significant effects at 100-fold Cmax. Among all 31 KIs tested, ceritinib caused the most significant cytochrome c release, that is, 33% of total cytochrome c was leaked after treatment, though this occurred at 100-fold Cmax (Fig. 5d).

### KI effects on ROS production in mitochondria

**Fig. 6:**
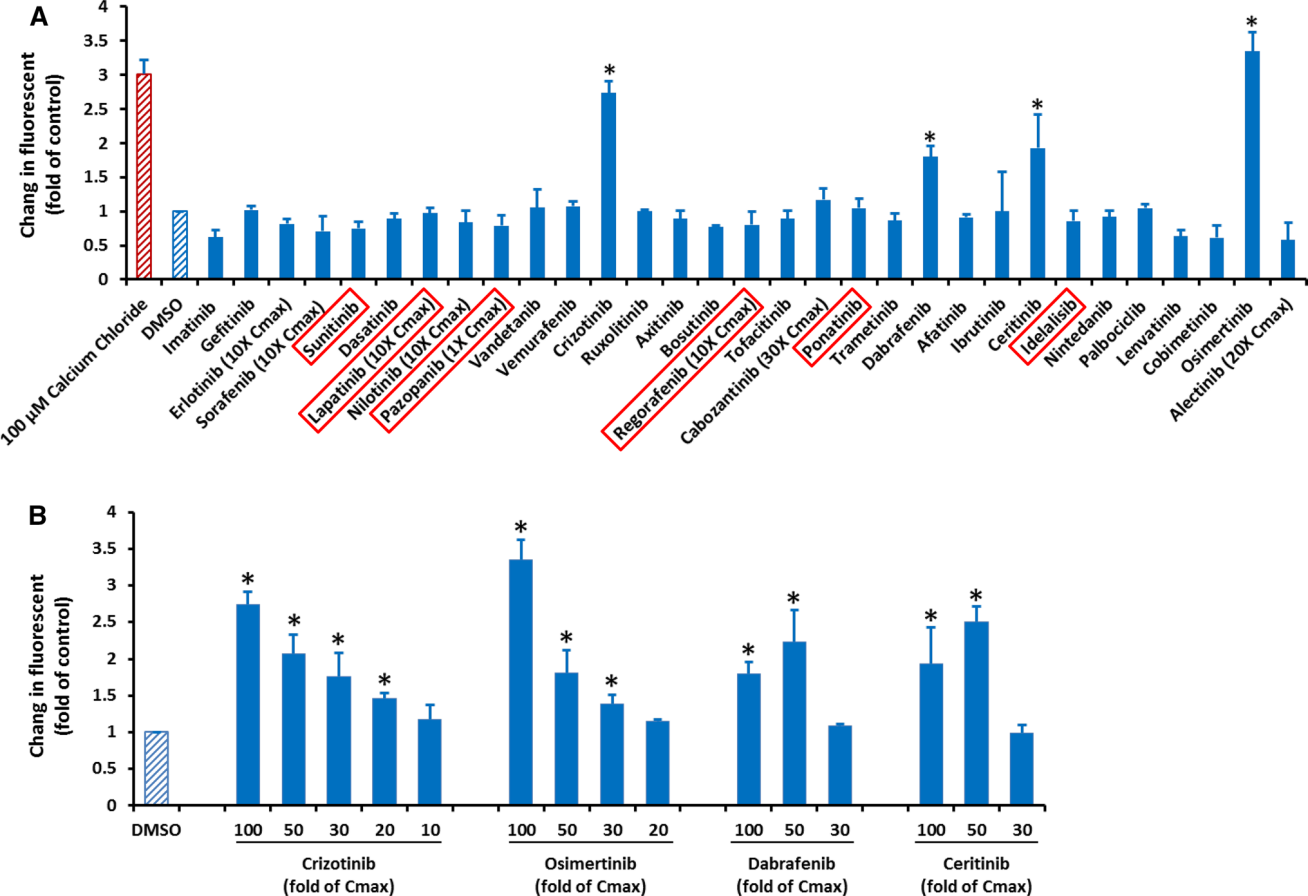
KI effects on ROS production in mitochondria. Rat liver mitochondria were loaded with 4 µM CM-H2DCFDA and then treated with KIs at 100-fold Cmax or the highest testable concentrations as indicated. Calcium chloride was used as a positive control. The fluorescence (excitation 490 nm, emission 530 nm) was measured every 1 min for 30 min. The signal from DMSO-treated samples was set as 1. The drugs in the *X*-axis were listed according to their approval date. The six KIs with a black box warning for hepatotoxicity were highlighted in a *red box* (**a**). **b** Shows the dose response of four KIs. Data are means and standard derivations from three independent experiments. *p < 0.05 as compared to DMSO-treated samples (color figure online)

ROS is produced at low levels during normal mitochondrial respiration. Drug-induced inhibition of oxygen consumption can lead to ROS overproduction. As shown in Fig. 6a, calcium chloride induced a threefold increase in ROS production. However, none of the KIs caused ROS over production in mitochondria at 1 to 10 fold Cmax (Supplementary Table 1). At the highest tested concentrations, four KIs including crizotinib, dabrafenib, ceritinib, and osimertinib caused significantly ROS production (Fig. 6a). The dose response of these four KIs is shown in Fig. 6b. While crizotinib and osimertinib trigger ROS overproduction beginning from 20-fold and 30-fold Cmax, respectively, dabrafenib and ceritinib only started to show significant effects from 50-fold Cmax. The magnitude of ROS production induced by osimertinib at 100-fold Cmax was even slightly higher than those caused by calcium chloride.

### Prediction of KI hepatotoxicity using mitotoxicity as compared to daily dose and Cmax

Table 5 shows the results of using KI mitotoxicity to predict hepatotoxicity. For this purpose, the drugs that cannot be tested at higher concentrations due to solubility issues will be either considered as mitotoxicity positive if they showed positive mitotoxicity at lower concentrations or excluded at untestable concentrations when low concentrations showed no mitotoxicity. At 100-fold Cmax with intact mitochondria, the PPV was 67% and NPV was only 31%, and the corresponding sensitivity and specificity was 53 and 44%, respectively. At 100-fold Cmax with both intact mitochondria and submitochondrial particles, though the specificity was not affect, other parameters were all increased, indicating that inclusion of submitochondrial particles enhanced the predictive power. At onefold Cmax with intact mitochondria, the PPV and specificity was all 100%, but NPV and sensitivity was only 32 and 13%, respectively. The results at other concentrations were presented in Supplementary Table 2. The overall results were that though the PPV was decent, the NPV was always no greater than 50%. Interestingly, using a cutoff of Cmax ≥ 1.1 µM (Shah et al. 2015) or daily dose ≥100 mg (Weng et al. 2015b), similar predictive results were obtained as using mitotoxicity data (Supplementary Table 2).

**Table 5:** Prediction of KI hepatotoxicity by using mitotoxicity at different concentrations

Fold of Cmax	Test system	Mitotoxicity	DILI+	DILI−	PPV	NPV	Sensitivity	Specificity
100	Intact-mito (excluded 3 drugs)	+	10	5	67%	31%	53%	44%
		−	9	4				
	Intact and Sub-mito (excluded 1 drug)	+	13	5	72%	33%	62%	44%
		−	8	4				
1	Intact-mito	+	3	0	100%	32%	14%	100%
		−	19	9				
	Intact and Sub-mito	+	3	0	100%	32%	14%	100%
		−	19	9				

*PPV* positive predictive value, *NPV* negative predictive value, *DILI* drug-induced liver injury. A drug is considered as mitotoxicity positive (+) if it causes at least one type of mitochondrial injury. Intact-mito: data from whole mitochondria; Sub-mito: data from submitochondrial particles

## Discussion

Though several KIs have been investigated in previous reports regarding mitochondrial toxicity, a comprehensive study involving all FDA approved KIs is not yet available. In addition, KI effects from previous reports cannot be compared directly, as different platforms were used. This study not only significantly expanded the number of KIs tested for mitochondrial toxicity, but also provided additional data on mitochondrial liabilities such as ROS production and RCC activities that were not examined in previous studies.

Some endpoints measured in this study can be related. For example, an uncoupler can disrupt inner membrane potential, and RCC inhibition may cause ROS overproduction, and cytochrome c release can affect oxygen consumption. However, the relation is not always causative. For example, the increase in ROS production caused by mild RCC inhibition can be antagonized by endogenous antioxidant. Another example is that RCC inhibition can decrease oxygen consumption and uncoupling can enhance oxygen consumption, and therefore if a KI is both a RCC inhibitor and an uncoupler, such as sorafenib, the net effects on oxygen consumption can be complicated. Further studies are needed to clarify the relations among mitochondrial changes caused by KIs.

Of the 31 KIs examined only three including sorafenib, regorafenib and pazopanib, all of which are DILI positive, caused significant mitochondrial toxicity at concentrations equal to the Cmax. At similar concentrations, sorafenib has been shown to be an uncoupler and inhibitor of OXPHOS in isolated rat heart mitochondria (Will et al. 2008) and regorafenib is reported to be a pure uncoupler without inhibition of OXPHOS in isolated rat liver mitochondria (Weng et al. 2015a), while no data are available for pazopanib. This study showed that sorafenib affected rat liver mitochondria in a similar manner as reported in rat heart mitochondria (Will et al. 2008). Sorafenib was also reported to be a strong mitochondrial toxicant in neuroblastoma cells (Bull et al. 2012). Collectively, these data suggest that sorafenib-induced effects on mitochondria are not tissue specific, and that mitochondrial toxicity may contribute to various types of organ toxicity associated with sorafenib. This study also identified an additional mitochondrial liability of regorafenib not reported in the previous study (Weng et al. 2015a), that is, regorafenib caused cytochrome c release at a concentration equal to Cmax. Of note is that the chemical structures of sorafenib and regorafenib are very similar (Supplementary Fig. 1). This study found that both drugs were uncouplers and both caused cytochrome c release, a decreased in MMP, and mitochondrial swelling, while neither of them affected ROS production. It appears likely that sorafenib and regorafenib share chemical properties that are associated with mitochondrial injury leading to organ toxicity.

The drug pazopanib, which carries a BBW for DILI on its labeling, has the highest Cmax (133 µM) among all KIs examined and it is not possible to test its effects at more than onefold Cmax concentrations due to the solubility issue. At onefold Cmax, pazopanib inhibited state 3 mitochondrial respiration driven by glutamate/malate by over 50%, but showed no effects on succinate-driven respiration, indicating that pazopanib may specifically inhibit RCC I. Indeed, with submitochondrial particles, pazopanib was confirmed to be an inhibitor of RCC I but not other RCCs. These data suggest that selective inhibition of RCC I may contribute to the pathogenesis of pazopanib hepatotoxicity.

The drugs ponatinib and sunitinib have BBWs for DILI in the labeling. However, both drugs showed no effects on intact mitochondria or submitochondrial particles even at the highest concentrations tested, indicating that mitochondrial liability at 100-Cmax would not predict DILI positive KI with high confidence. Alternative mechanisms, such as reactive metabolites, may account for DILI associated with these two drugs. Our findings on sunitinib with intact mitochondria are consistent with previous reports carried out either in rat heart mitochondria (Will et al. 2008) or mouse liver mitochondria (Porceddu et al. 2012). However, in the latter mouse study, sunitinib was considered as mitochondrial toxicity positive, as the authors determined that the Cmax of sunitinib was 70.8 µM (Porceddu et al. 2012), which is sharp contrast with ours (0.12 µM) and others’ (0.25 µM) (Will et al. 2008). If the true Cmax of sunitinib was 70.8 µM, it would not be feasible for us to have it tested at 100-fold Cmax due to the solubility issue.

The DILI negative drug vandetanib inhibited RCC I starting at 50-fold Cmax, but had no effects on mitochondrial oxygen consumption even at 100-fold Cmax. The likely reason for this discrepancy is that RCC activities need to be measured using submitochondrial particles, while oxygen consumption is determined using intact mitochondria. Differential effects of chemicals on whole mitochondria and submitochondrial particles have been reported previously, and one likely reason, among many others, was that the compound may not easily cross the inner membrane and therefore RCC proteins were protected from the drug’s effects with intact mitochondria (Weng et al. 2014). Further investigations are needed to confirm if this is true for vandetanib and other KIs showing similar effects. On the other hand, another DILI negative drug dabrafenib caused significant inhibition of state 3 respiration in intact mitochondria but showed no effects on RCC activities in submitochondrial particles. The likely reason for this discrepancy is that dabrafenib also caused cytochrome c release leading to suppressed oxygen consumption. It should be pointed out that these two DILI negative drugs indeed caused various types of mitochondrial injury at 100-fold Cmax, implying that mitochondrial liability at 100-Cmax is not a good predictor of DILI negative KIs. Further supporting this premise, the DILI negative drugs dabrafenib, cabozantinib, ruxolitinib, osimertinib, and ibrutinib also showed significant toxicity to intact mitochondria at concentrations starting from 5-fold, 10-fold, 20-fold, 30-fold, and 50-fold Cmax, respectively. Interestingly, one of the veterinary KIs oclacitinib has not been associated with DILI in animals. However, at 50-fold Cmax, oclacitinib caused significant cytochrome c release from intact mitochondria (data not shown). Taken together, these data indicate that mitochondrial toxicity was too sensitive in predicting DILI negative KIs. However, the possibility that the DILI potential of these KIs has not been fully recognized from clinical use cannot be ruled out.

The predictive power of the mitochondrial model for KI hepatotoxicity presented here is in contrast to a previous report showing that human DILI in general can be predicted with high confidence using in vitro mitochondrial endpoints measured at 100-fold Cmax (Porceddu et al. 2012). The likely reason for this discrepancy is that KI hepatotoxicity may differ from DILI caused by other therapeutic groups of drugs. To our knowledge, though several in vitro systems have been proposed to be effective in predicting a compound’s DILI potential, no previous studies have included all FDA approved KIs for this purpose. Our findings highlight the challenge posed by KIs for predicting DILI using mitochondrial assays and warrant further investigations using alternative in vitro systems.

An interesting observation is that mitochondrial toxicity at onefold and 2.5-fold Cmax showed 100% PPV and 100% specificity in predicting KI hepatotoxicity, though the NPV and sensitivity were 32 and 14%, respectively. This indicates that if a drug causes mitotoxicity in any of the endpoints assessed at concentrations equal to Cmax, it will very likely be DILI positive; however, a drug being non-mitotoxic at Cmax does not mean that it will be DILI negative, as alternative or indirect mechanisms may be involved. It appears that mitotoxicity at 1–2.5-fold Cmax can be used to help identify hepatotoxic KIs as an early safety screen during drug discovery.

Many drugs impair mitochondrial functions contributing to DILI pathogenesis, although only a few have been directly tested in vivo or in humans (McGill et al. 2012). Drugs affecting mitochondrial functions in vitro at clinically relevant concentrations are expected to have a high likelihood of doing so under in vivo conditions. This study identified several KIs causing significant mitochondrial injury at concentrations equal to Cmax or only at several fold of Cmax. These drugs can serve as good candidates for future exploratory in vivo studies. Mitochondrion-enriched molecules, such as mitochondrial DNA and cytochrome c, can be released into the blood when liver injury is induced by drugs causing mitochondrial damage, and circulating mitochondrial molecules have been shown to be novel biomarkers for DILI induced by mitotoxic drugs (Miller et al. 2008; Shi et al. 2015). It is worthwhile to examine if KI hepatotoxicity can be monitored using circulating mitochondrial biomarkers. The KIs that showed the strongest mitochondrial toxicities, such as sorafenib, regorafenib, and pazopanib, are particularly suitable for testing this hypothesis in future investigations.

It should be pointed out that the DILI potential of some drugs was not fully recognized until being marketed for many years (Rivkees 2010). As most KIs were approved very recently, the DILI potential of these drugs may not be thoroughly understood until more time has passed. Therefore, the predictive results presented here may change over time as more patients take these drugs. Nevertheless, our findings demonstrate that mitochondrial toxicity has limited predictive power for human DILI observed in clinical trials and post-marketing, studies that laid the foundation for drug approval and labeling. Alternative in vitro approaches, such as primary hepatocytes or human-induced pluripotent stem cell-derived hepatocytes, may partially overcome key disadvantages of isolated mitochondria, such as the lack of major drug metabolizing enzymes or regeneration/compensatory mechanisms. Given that human DILI remains poorly predicted by animal models, due possibly to the polymorphic nature of the human liver, further investigations into such in vitro approaches with addition of drug metabolizing enzymes or use of more human-based systems may likely prove worthwhile in future studies.

## Electronic supplementary material

Below is the link to the electronic supplementary material.

Supplementary material 1 (PPTX 1445 kb)

Supplementary material 2 (PPTX 115 kb)

Supplementary material 3 (XLSX 30 kb)

Supplementary material 4 (XLSX 14 kb)
