# Supplementary material for: Effects of 31 FDA approved small-molecule kinase inhibitors on isolated rat liver mitochondria
Source: Arch Toxicol. 2016 Dec 28;91(8):2921–38. doi: 10.1007/s00204-016-1918-1 (PMC5515969; doi:10.1007/s00204-016-1918-1)
Supplement: Supplementary file 1 — Supplementary material 1 (PPTX 1445 kb) [file 204_2016_1918_MOESM1_ESM.pptx]

## Slide 1
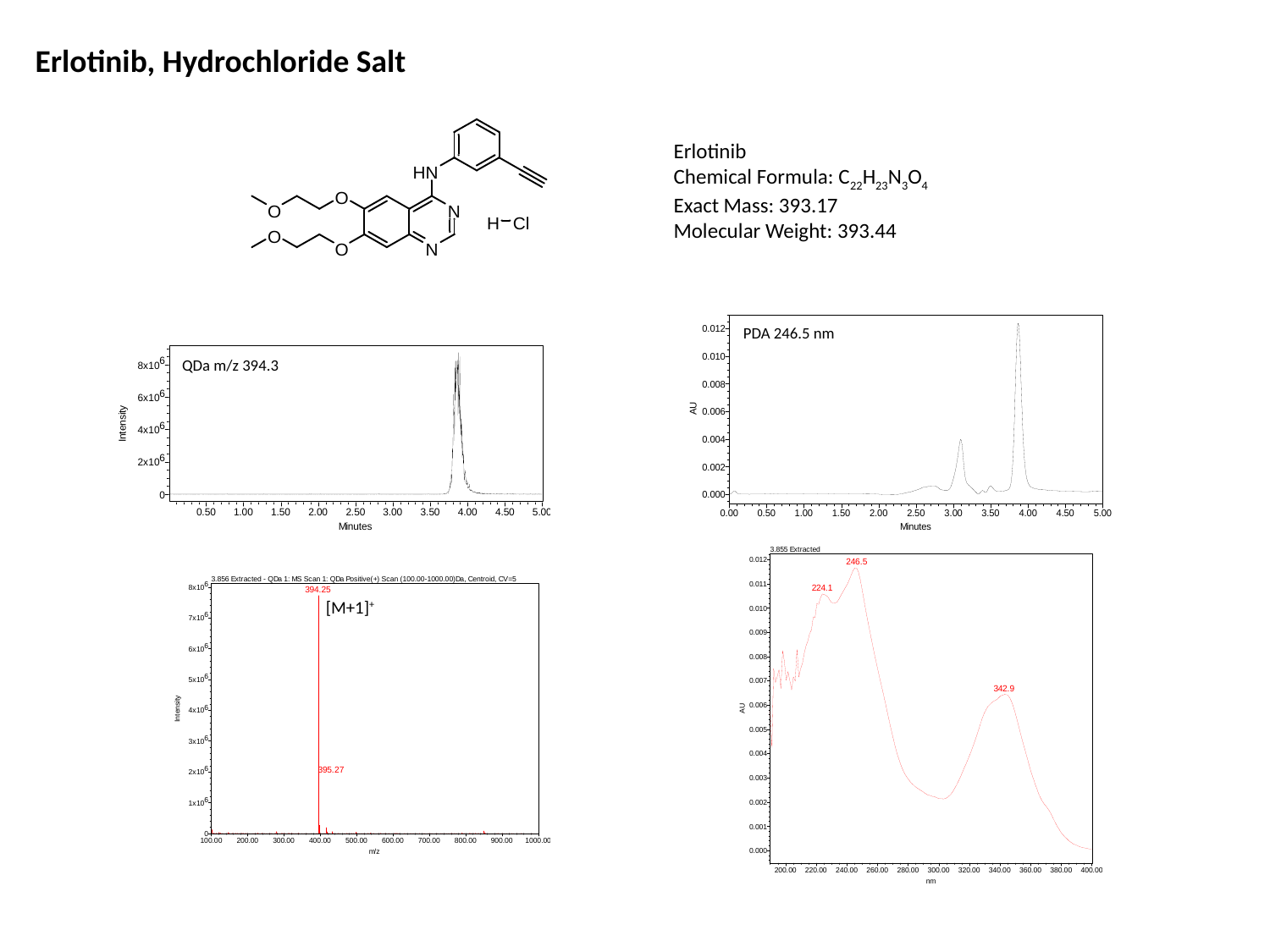

Erlotinib, Hydrochloride Salt
Erlotinib
Chemical Formula: C22H23N3O4
Exact Mass: 393.17
Molecular Weight: 393.44
PDA 246.5 nm
QDa m/z 394.3
[M+1]+

## Slide 2
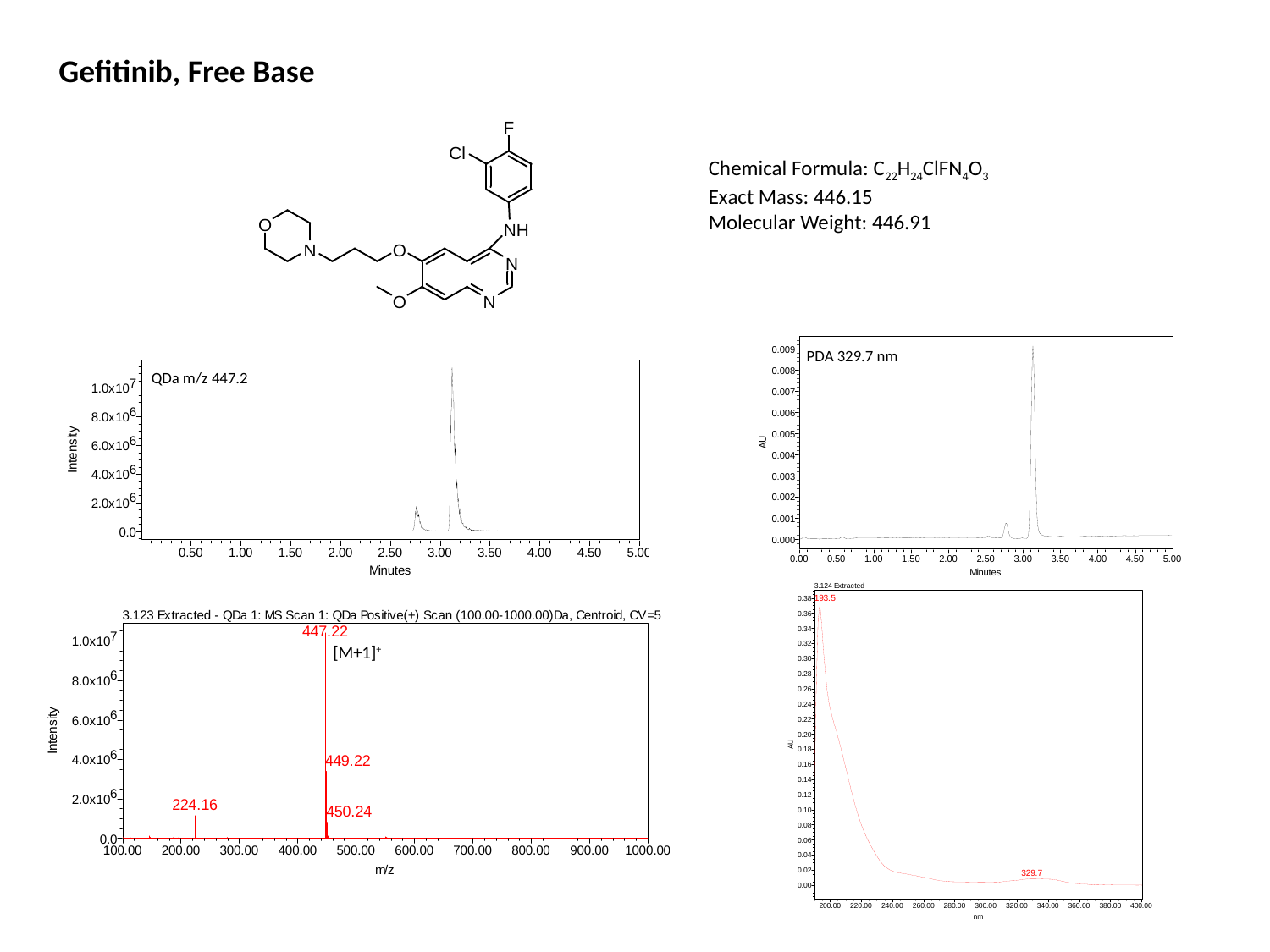

Gefitinib, Free Base
Chemical Formula: C22H24ClFN4O3
Exact Mass: 446.15
Molecular Weight: 446.91
PDA 329.7 nm
QDa m/z 447.2
[M+1]+

## Slide 3
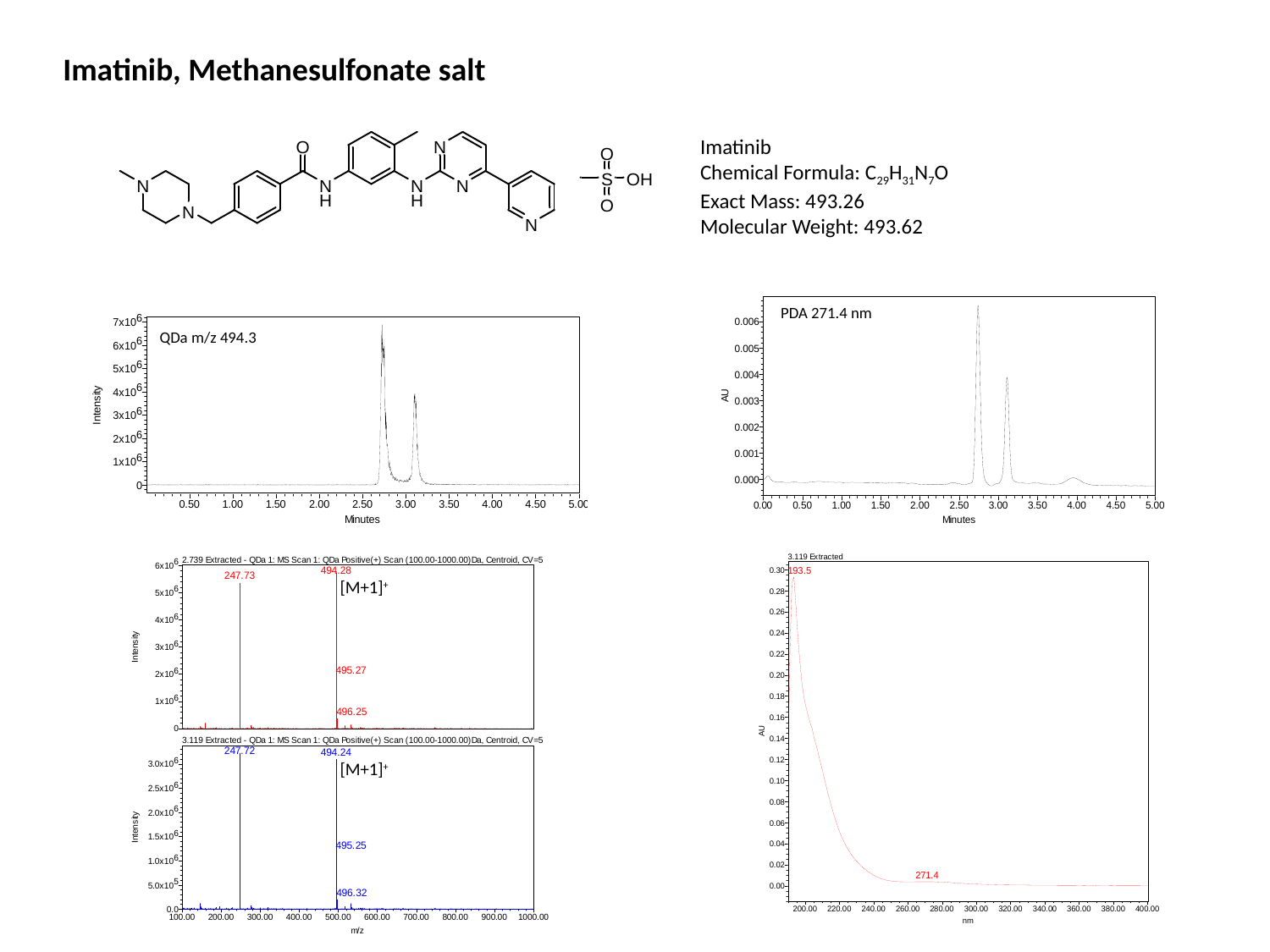

Imatinib, Methanesulfonate salt
Imatinib
Chemical Formula: C29H31N7O
Exact Mass: 493.26
Molecular Weight: 493.62
PDA 271.4 nm
QDa m/z 494.3
[M+1]+
[M+1]+

## Slide 4
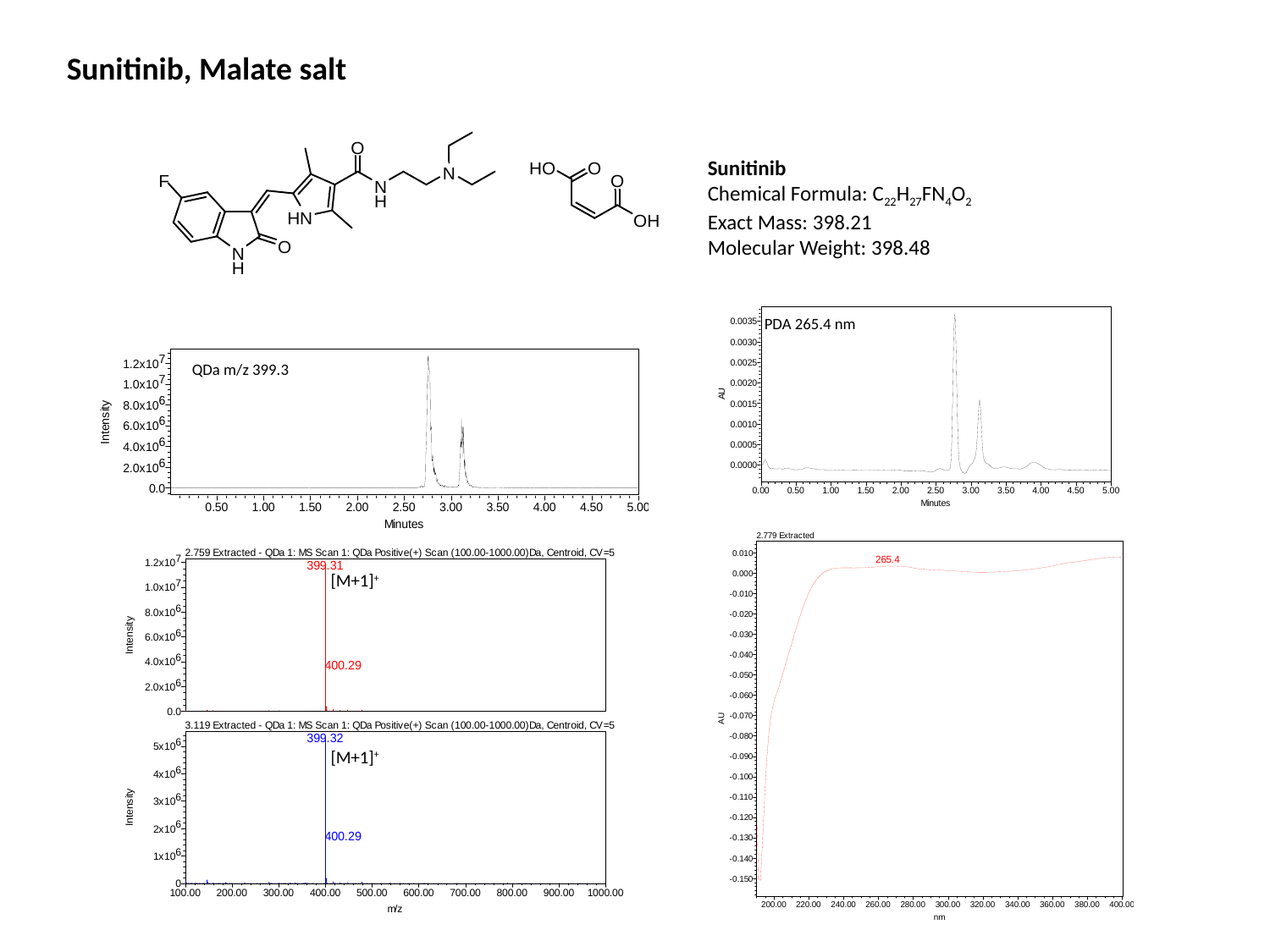

Sunitinib, Malate salt
Sunitinib
Chemical Formula: C22H27FN4O2
Exact Mass: 398.21
Molecular Weight: 398.48
PDA 265.4 nm
QDa m/z 399.3
[M+1]+
[M+1]+

## Slide 5
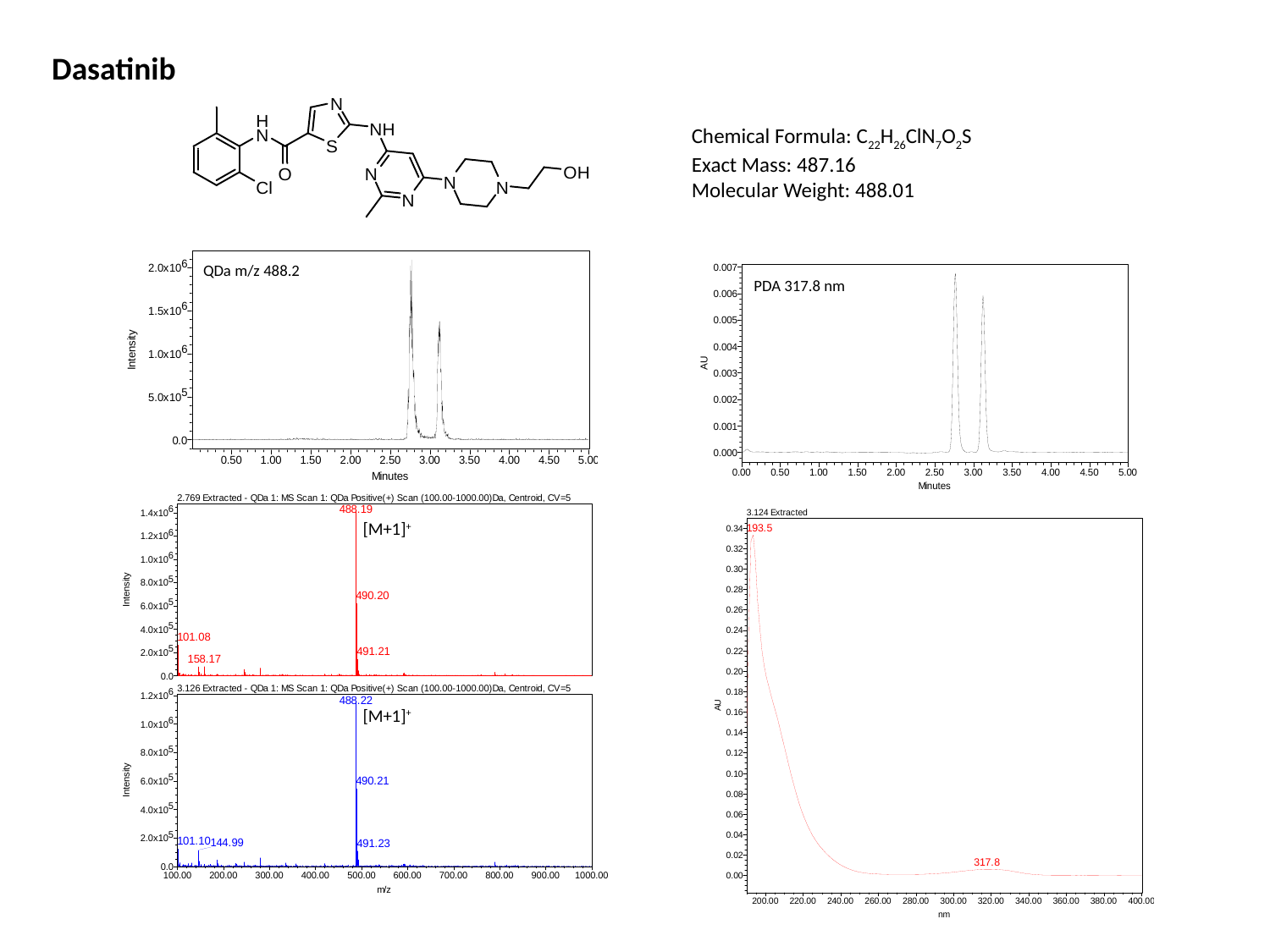

Dasatinib
Chemical Formula: C22H26ClN7O2S
Exact Mass: 487.16
Molecular Weight: 488.01
QDa m/z 488.2
PDA 317.8 nm
[M+1]+
[M+1]+

## Slide 6
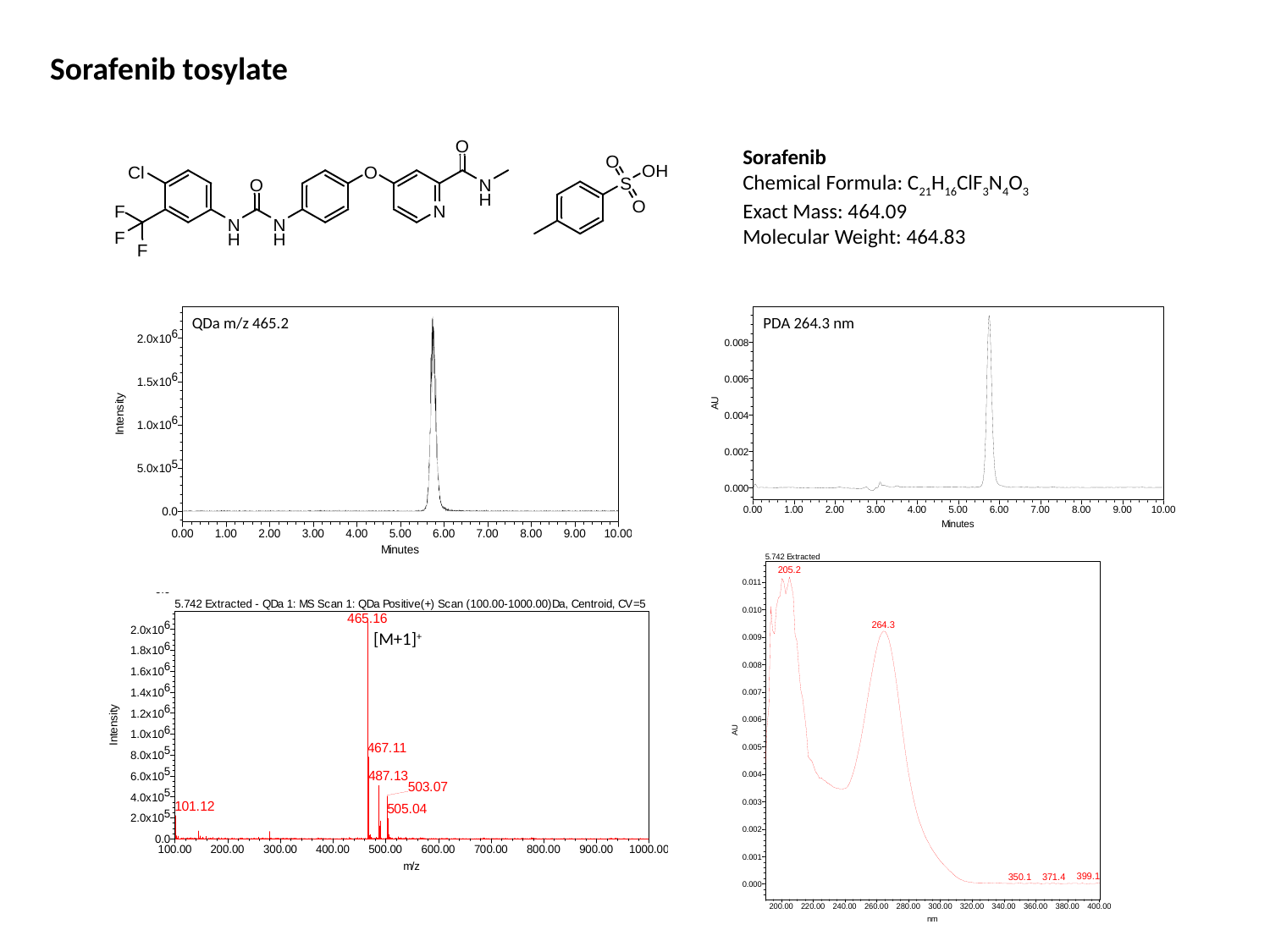

Sorafenib tosylate
Sorafenib
Chemical Formula: C21H16ClF3N4O3
Exact Mass: 464.09
Molecular Weight: 464.83
QDa m/z 465.2
PDA 264.3 nm
[M+1]+

## Slide 7
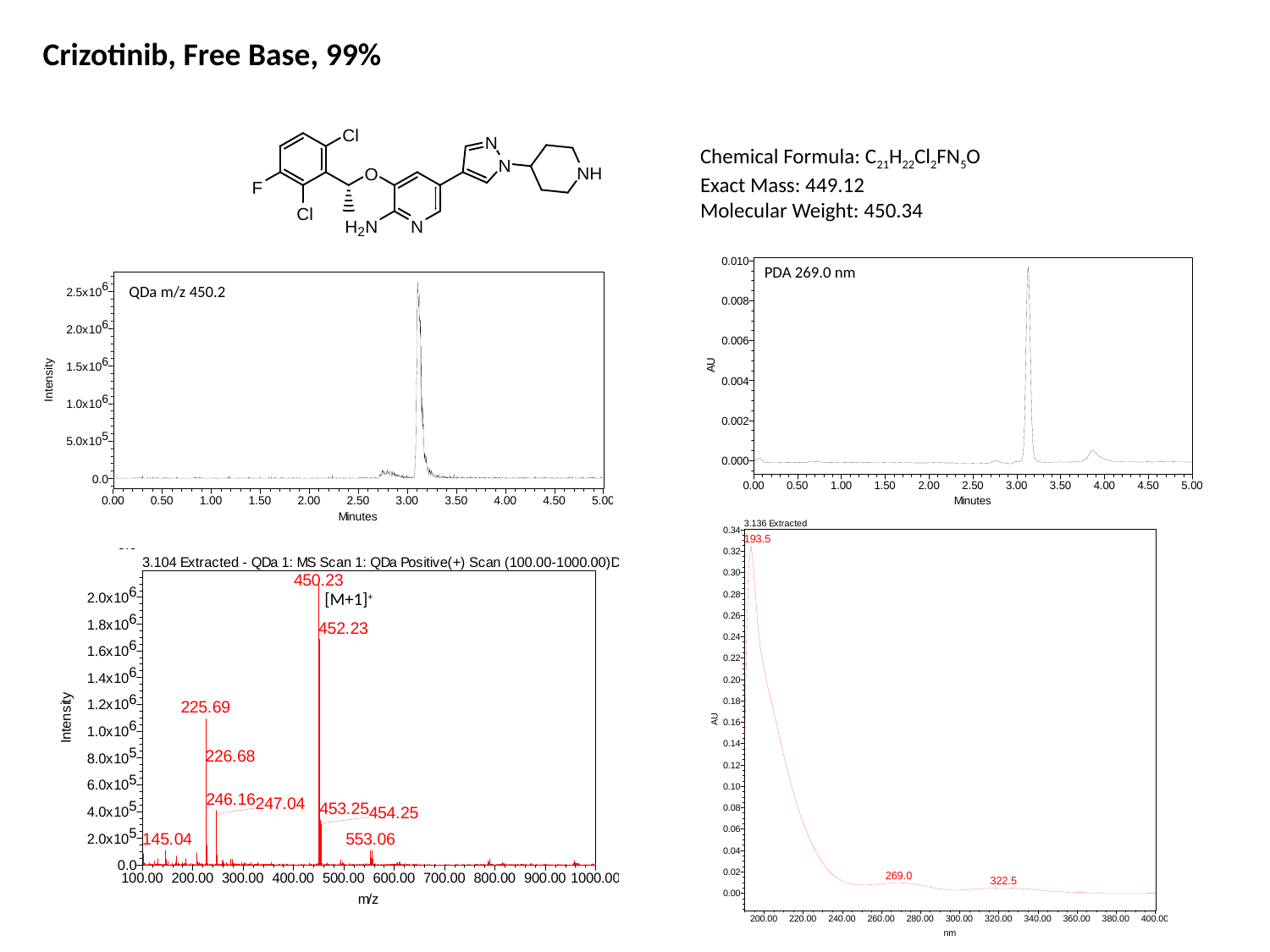

Crizotinib, Free Base, 99%
Chemical Formula: C21H22Cl2FN5O
Exact Mass: 449.12
Molecular Weight: 450.34
PDA 269.0 nm
QDa m/z 450.2
[M+1]+

## Slide 8
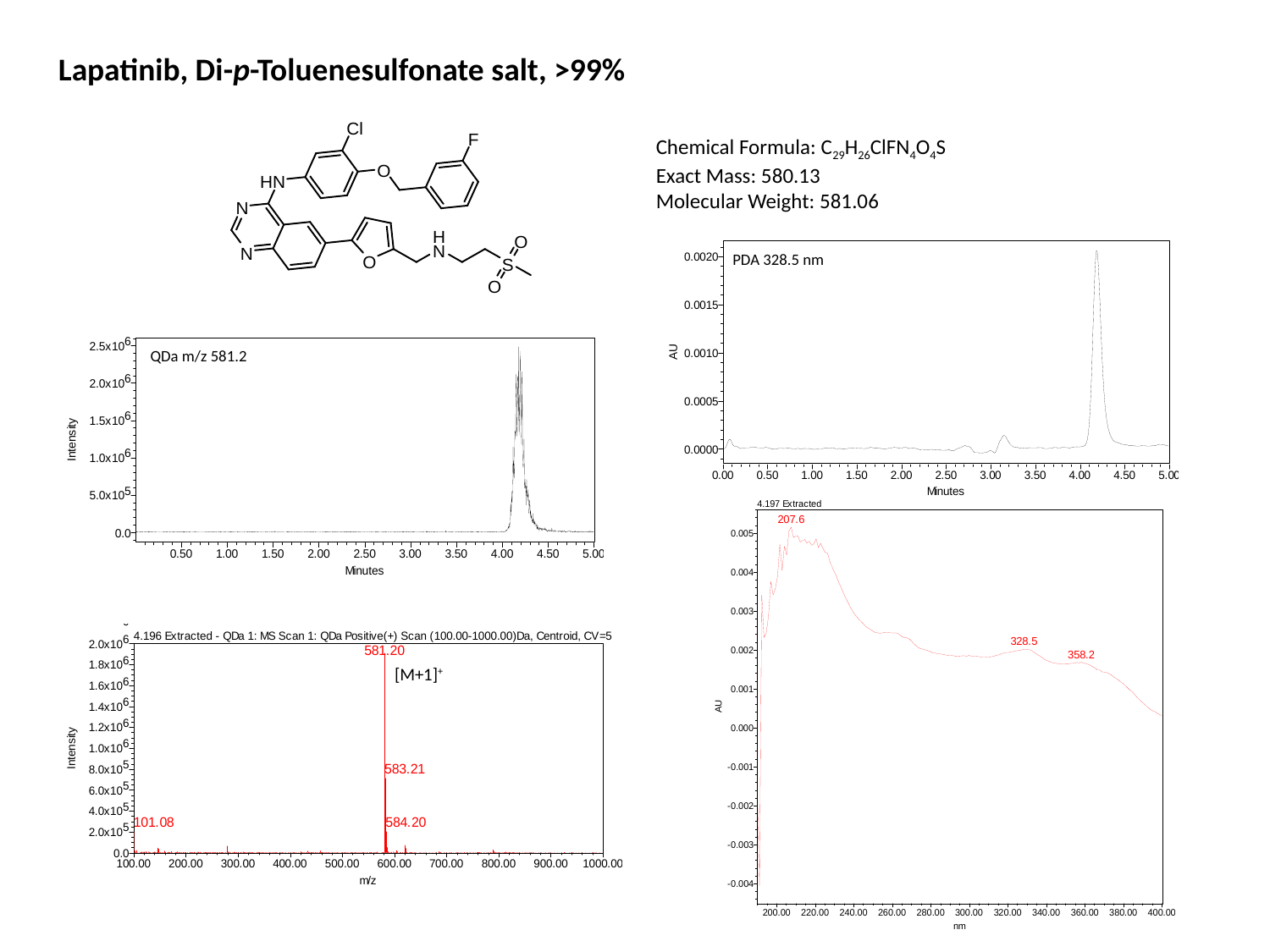

Lapatinib, Di-p-Toluenesulfonate salt, >99%
Chemical Formula: C29H26ClFN4O4S
Exact Mass: 580.13
Molecular Weight: 581.06
PDA 328.5 nm
QDa m/z 581.2
[M+1]+

## Slide 9
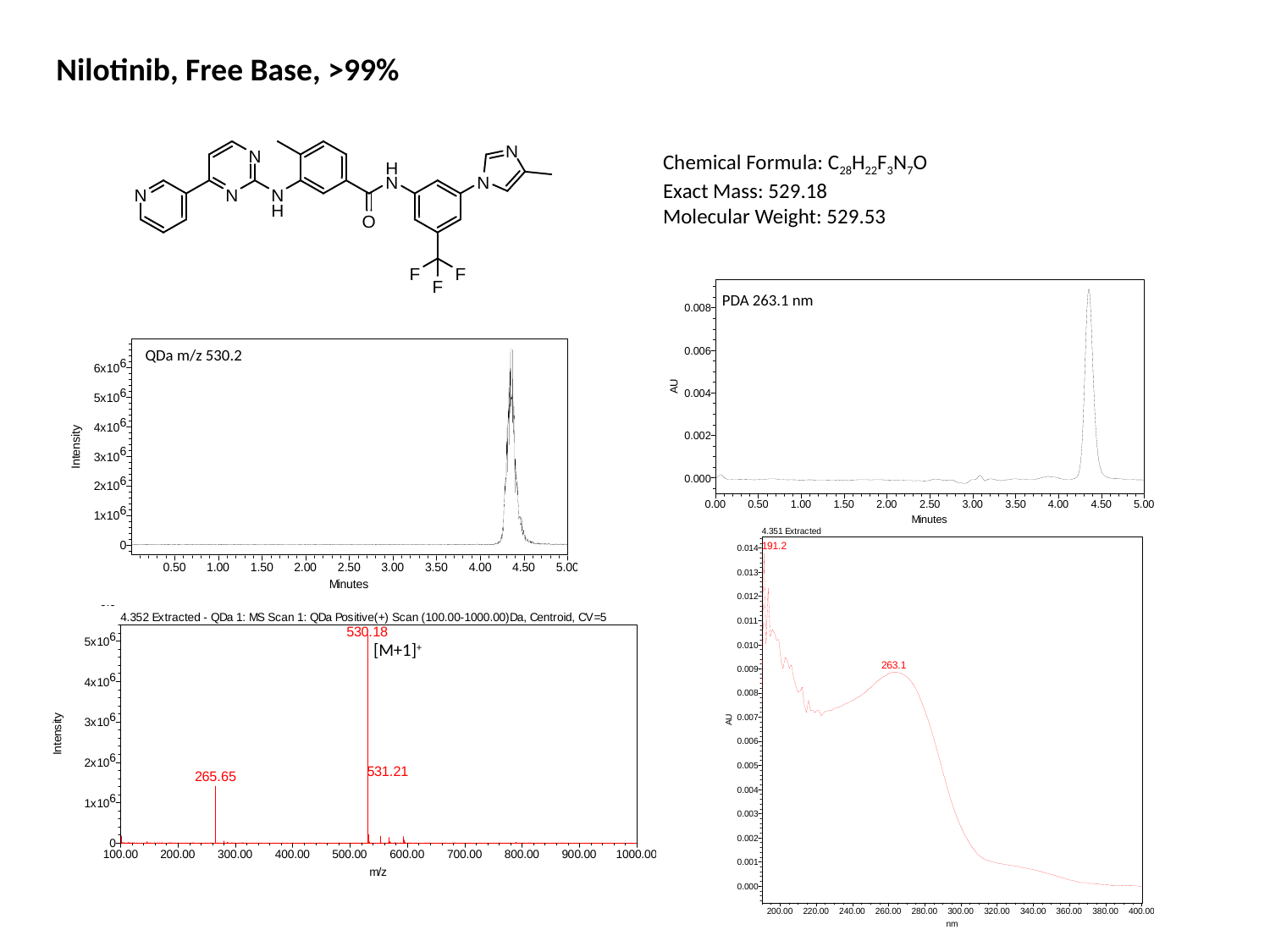

Nilotinib, Free Base, >99%
Chemical Formula: C28H22F3N7O
Exact Mass: 529.18
Molecular Weight: 529.53
PDA 263.1 nm
QDa m/z 530.2
[M+1]+

## Slide 10
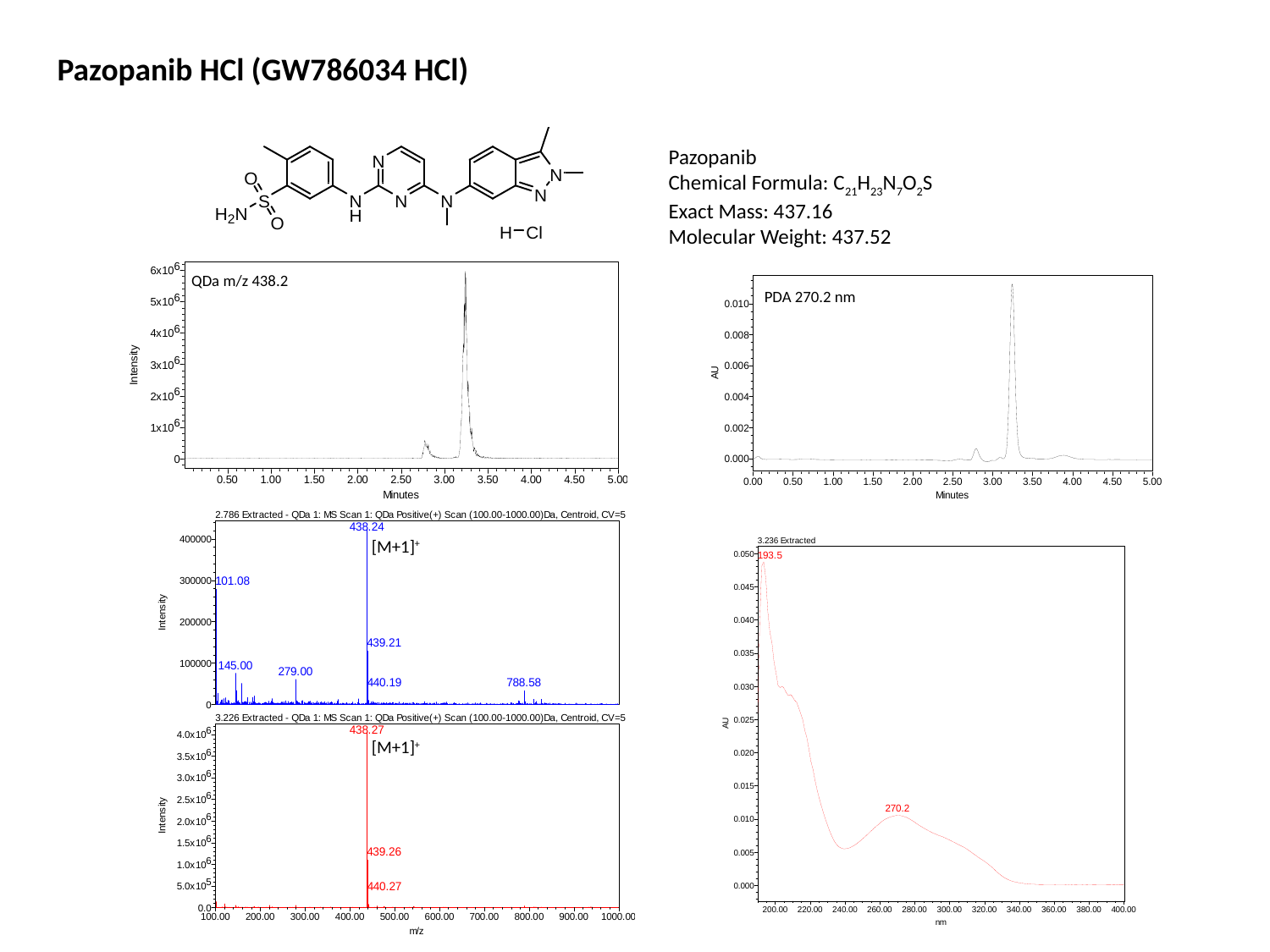

Pazopanib HCl (GW786034 HCl)
Pazopanib
Chemical Formula: C21H23N7O2S
Exact Mass: 437.16
Molecular Weight: 437.52
QDa m/z 438.2
PDA 270.2 nm
[M+1]+
[M+1]+

## Slide 11
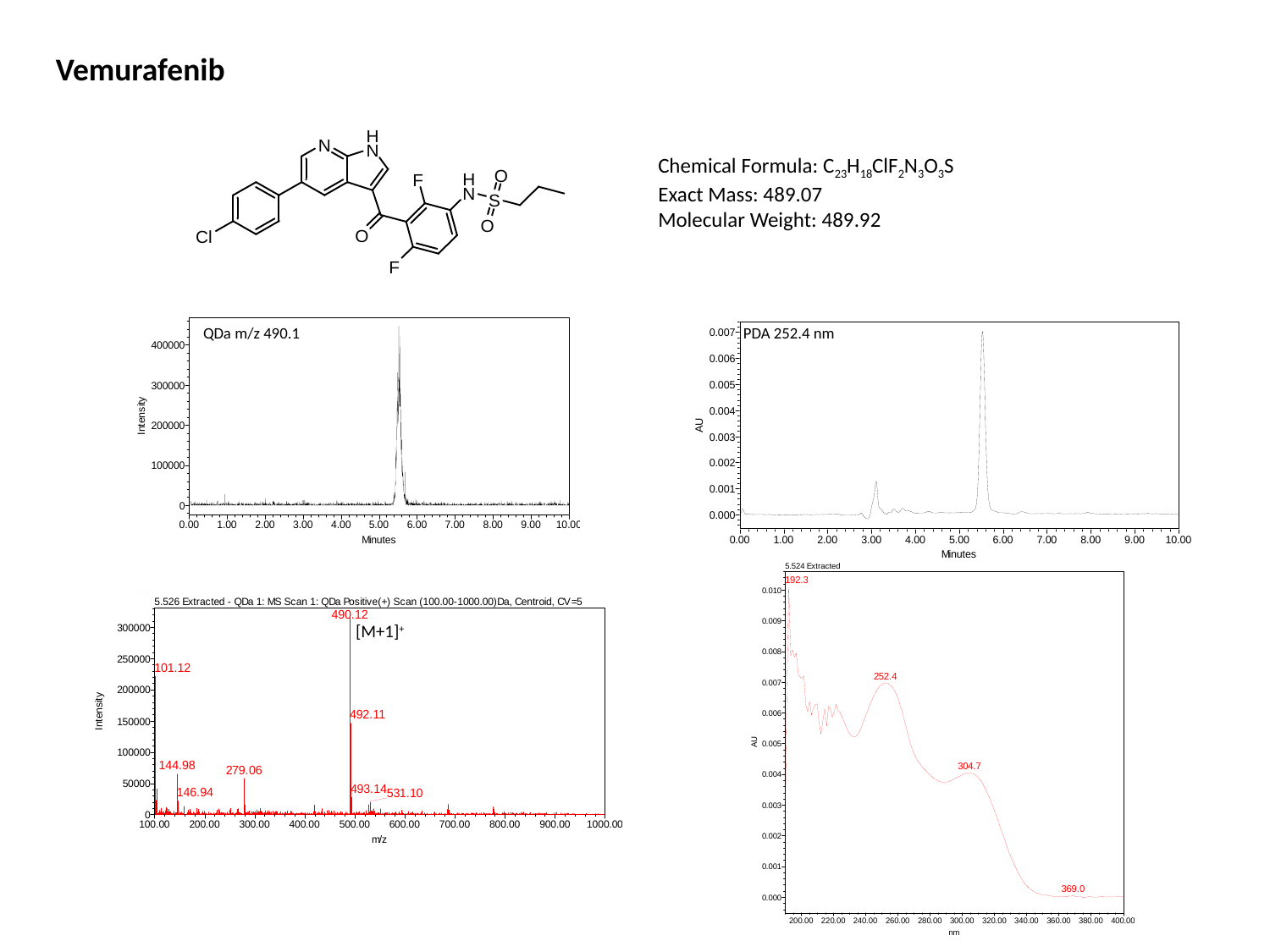

Vemurafenib
Chemical Formula: C23H18ClF2N3O3S
Exact Mass: 489.07
Molecular Weight: 489.92
QDa m/z 490.1
PDA 252.4 nm
[M+1]+

## Slide 12
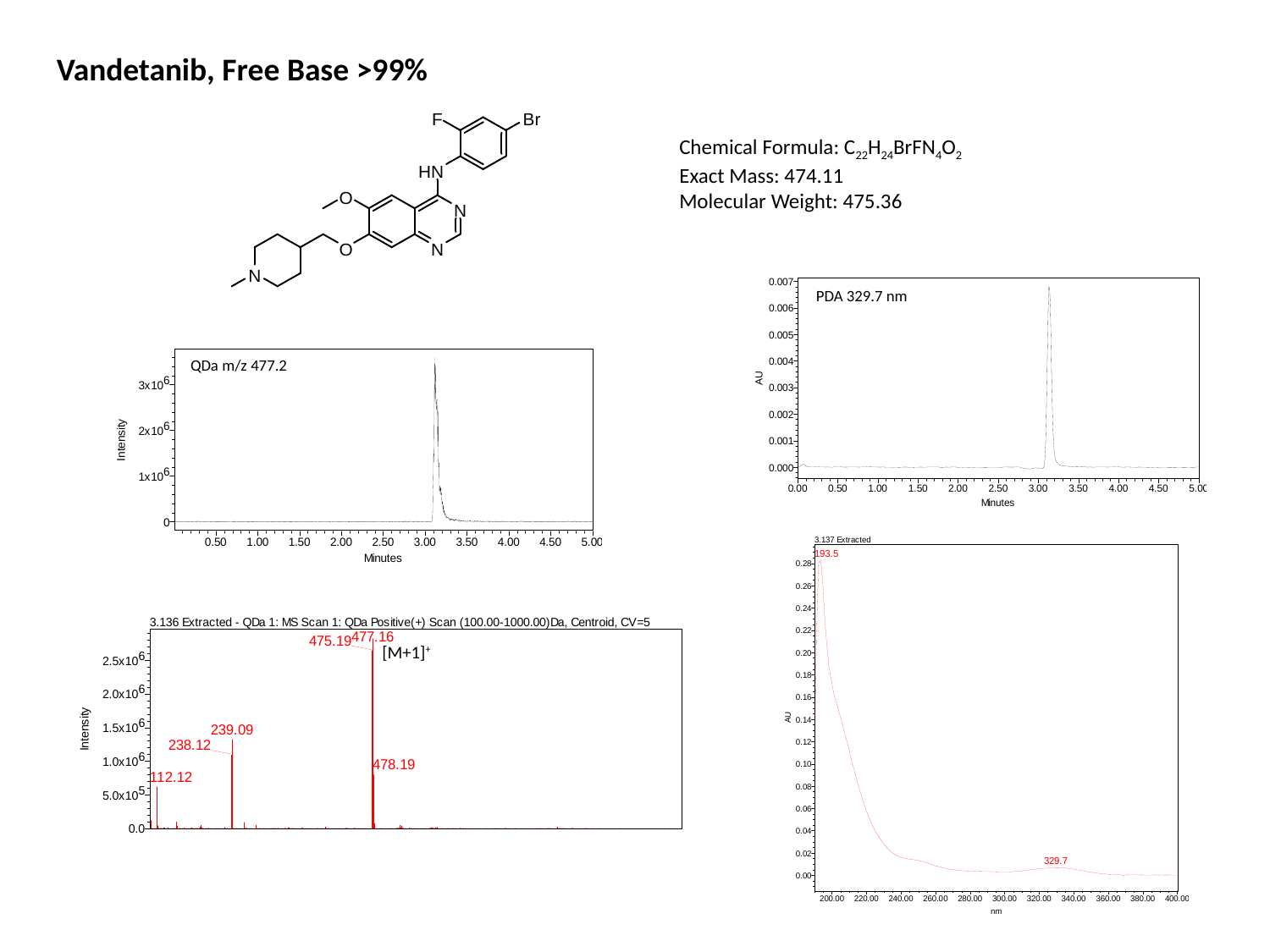

Vandetanib, Free Base >99%
Chemical Formula: C22H24BrFN4O2
Exact Mass: 474.11
Molecular Weight: 475.36
PDA 329.7 nm
QDa m/z 477.2
[M+1]+

## Slide 13
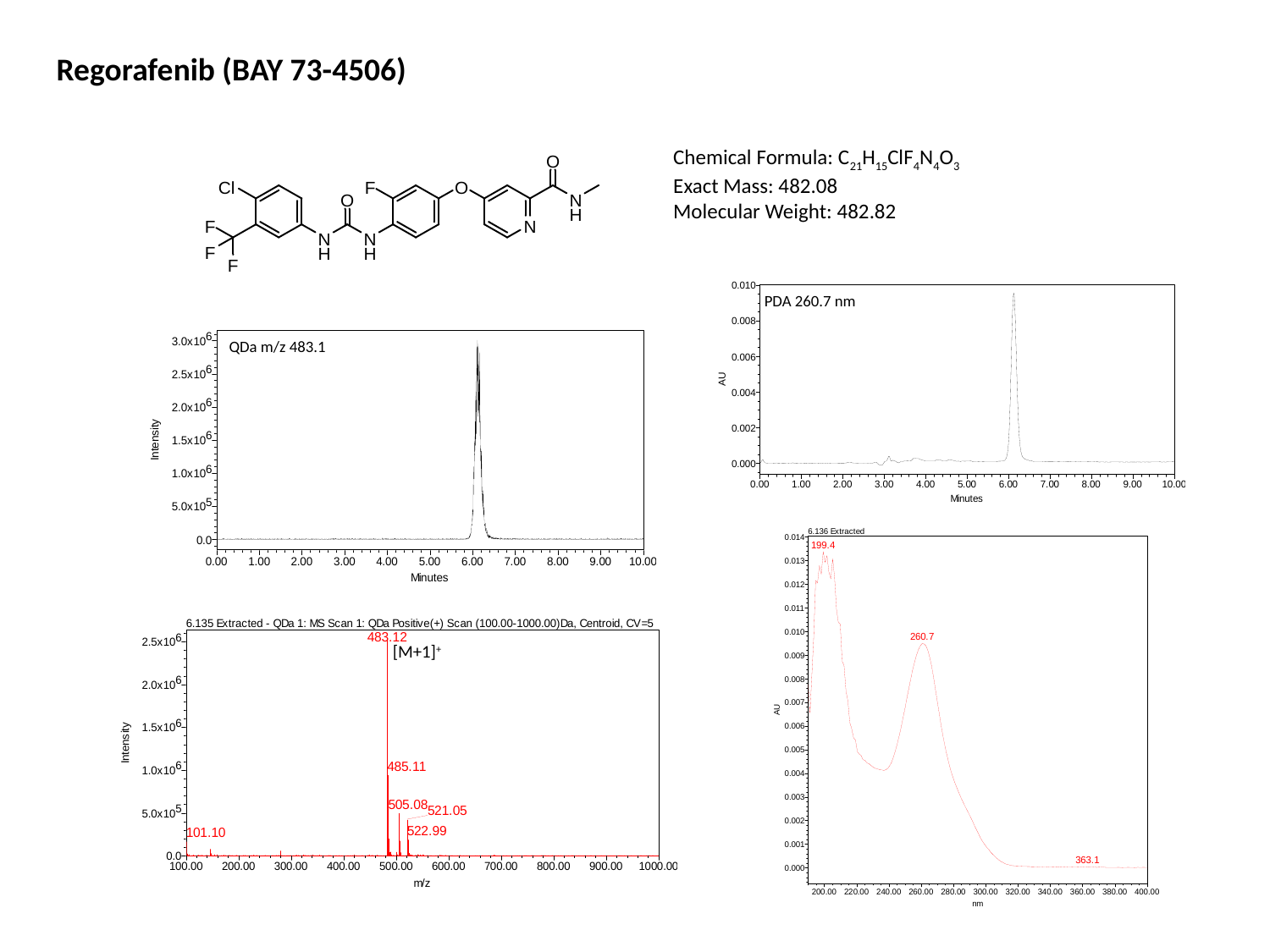

Regorafenib (BAY 73-4506)
Chemical Formula: C21H15ClF4N4O3
Exact Mass: 482.08
Molecular Weight: 482.82
PDA 260.7 nm
QDa m/z 483.1
[M+1]+

## Slide 14
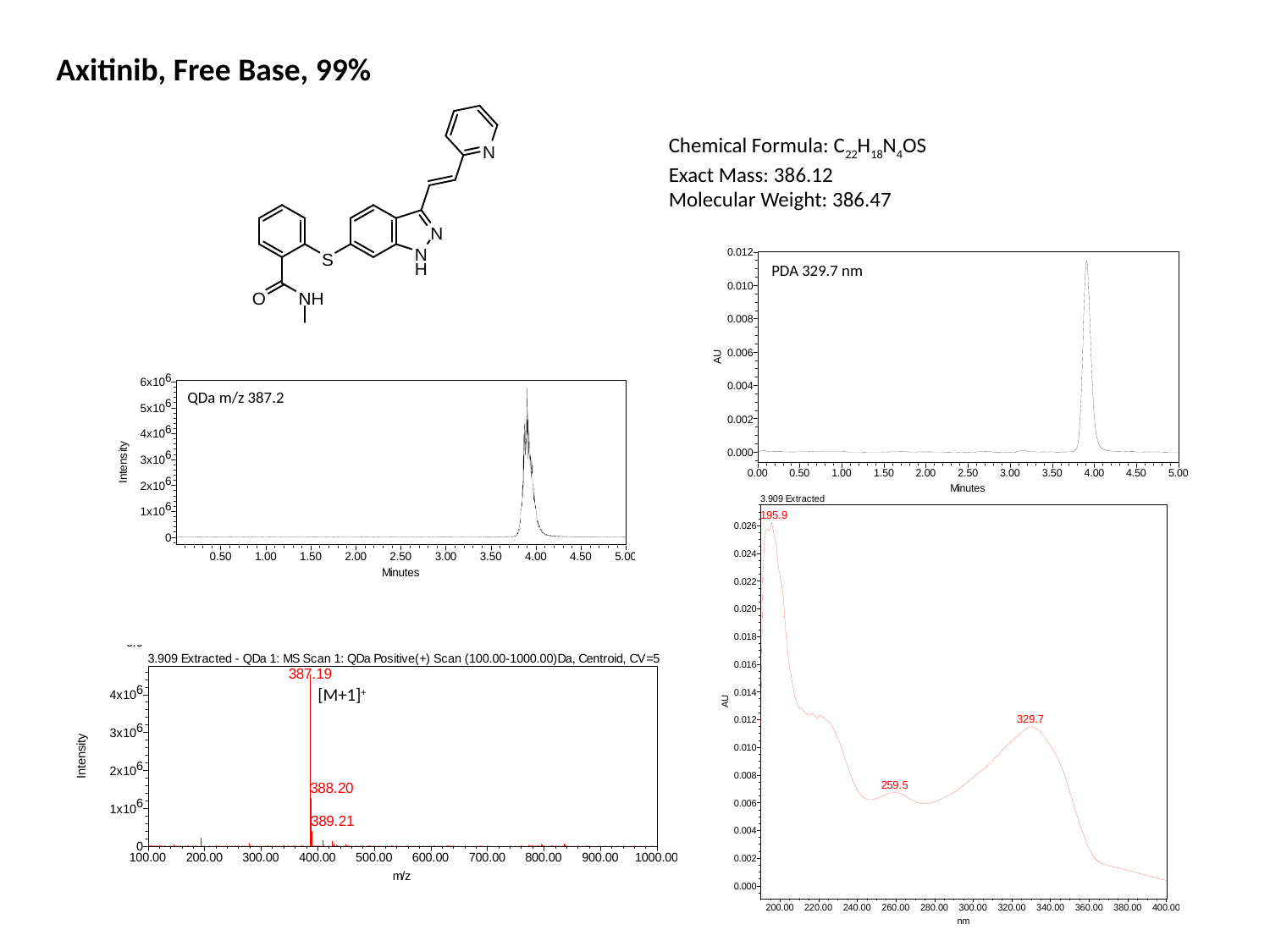

Axitinib, Free Base, 99%
Chemical Formula: C22H18N4OS
Exact Mass: 386.12
Molecular Weight: 386.47
PDA 329.7 nm
QDa m/z 387.2
[M+1]+

## Slide 15
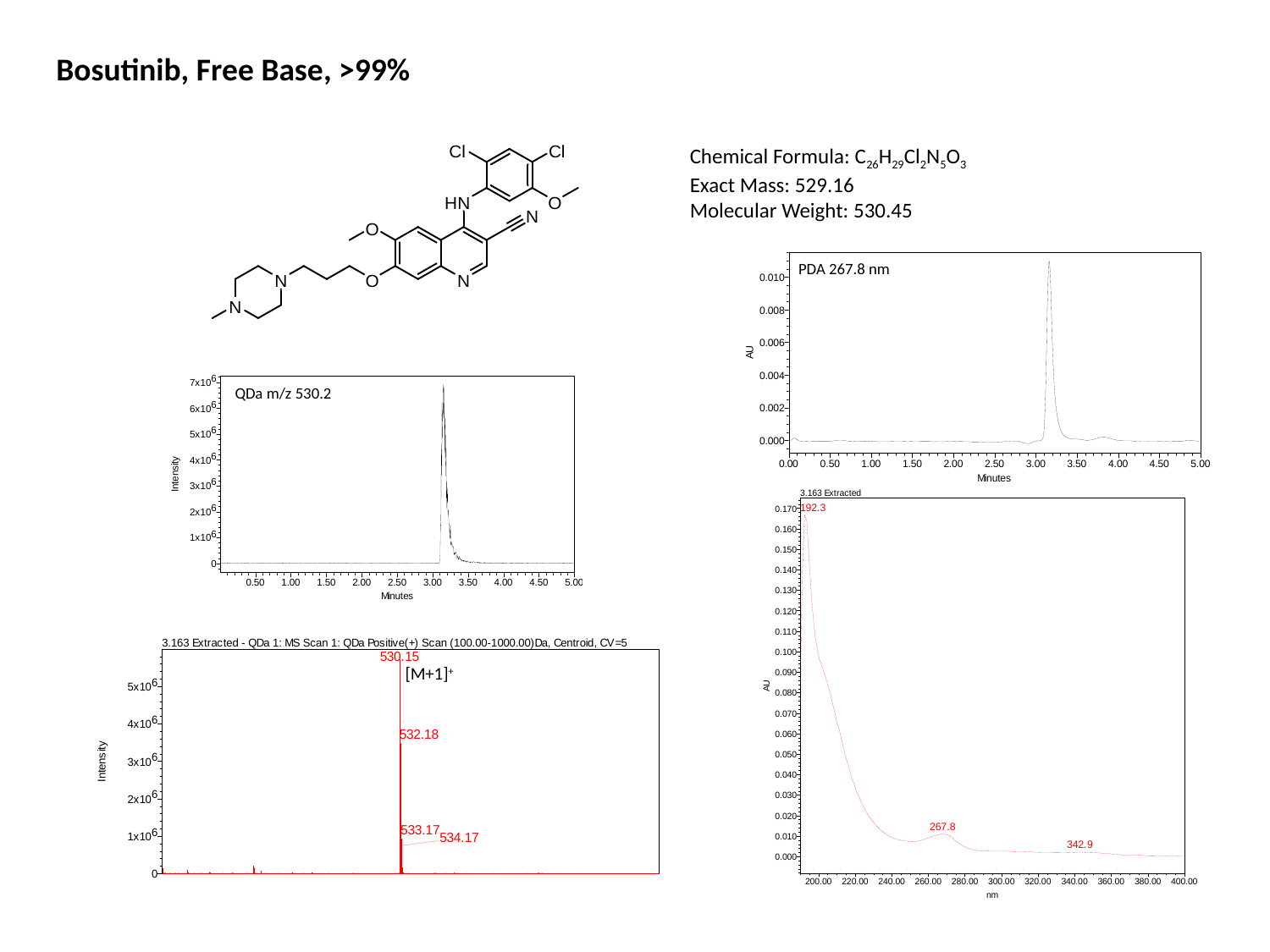

Bosutinib, Free Base, >99%
Chemical Formula: C26H29Cl2N5O3
Exact Mass: 529.16
Molecular Weight: 530.45
PDA 267.8 nm
QDa m/z 530.2
[M+1]+

## Slide 16
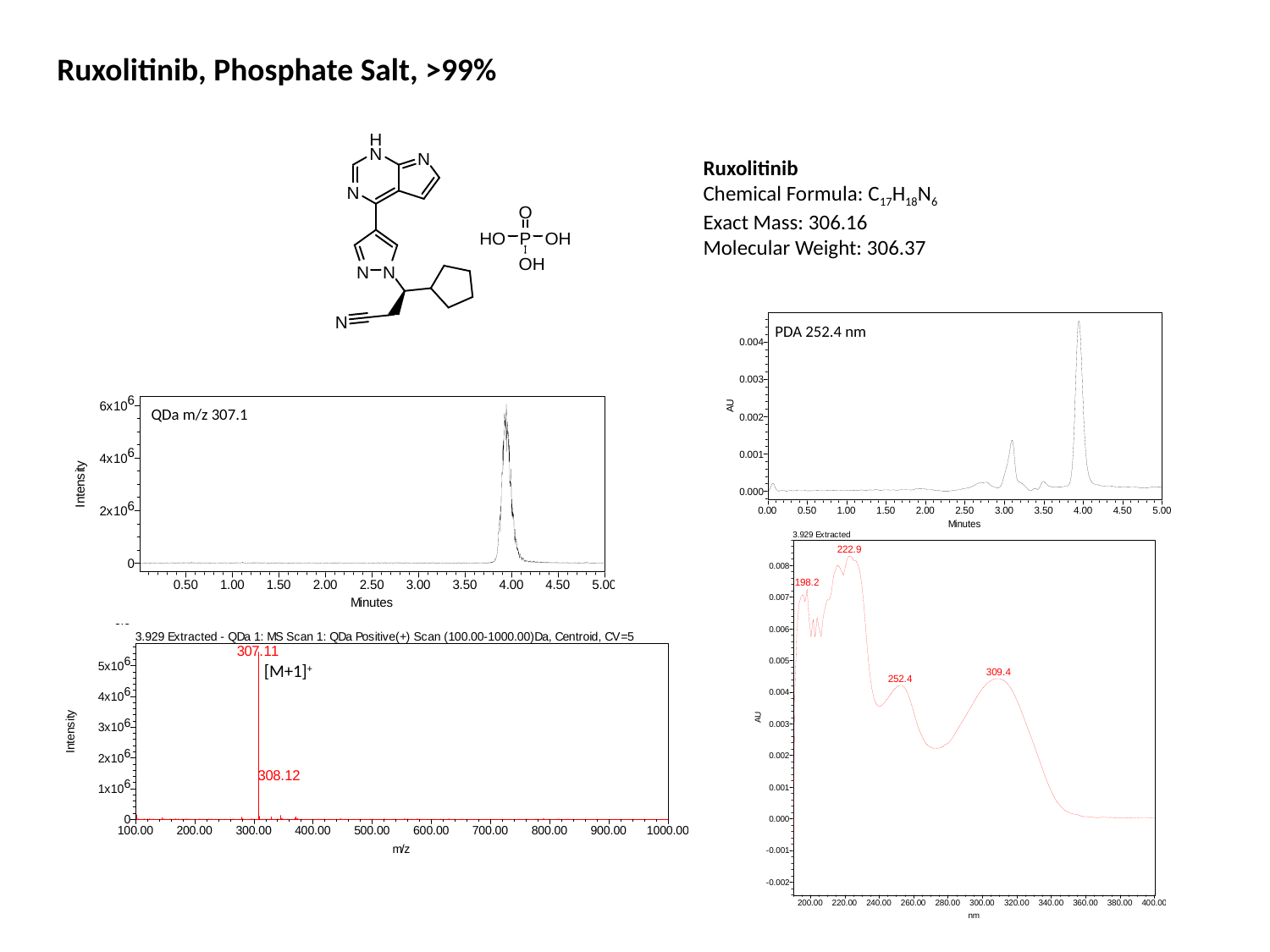

Ruxolitinib, Phosphate Salt, >99%
Ruxolitinib
Chemical Formula: C17H18N6
Exact Mass: 306.16
Molecular Weight: 306.37
PDA 252.4 nm
QDa m/z 307.1
[M+1]+

## Slide 17
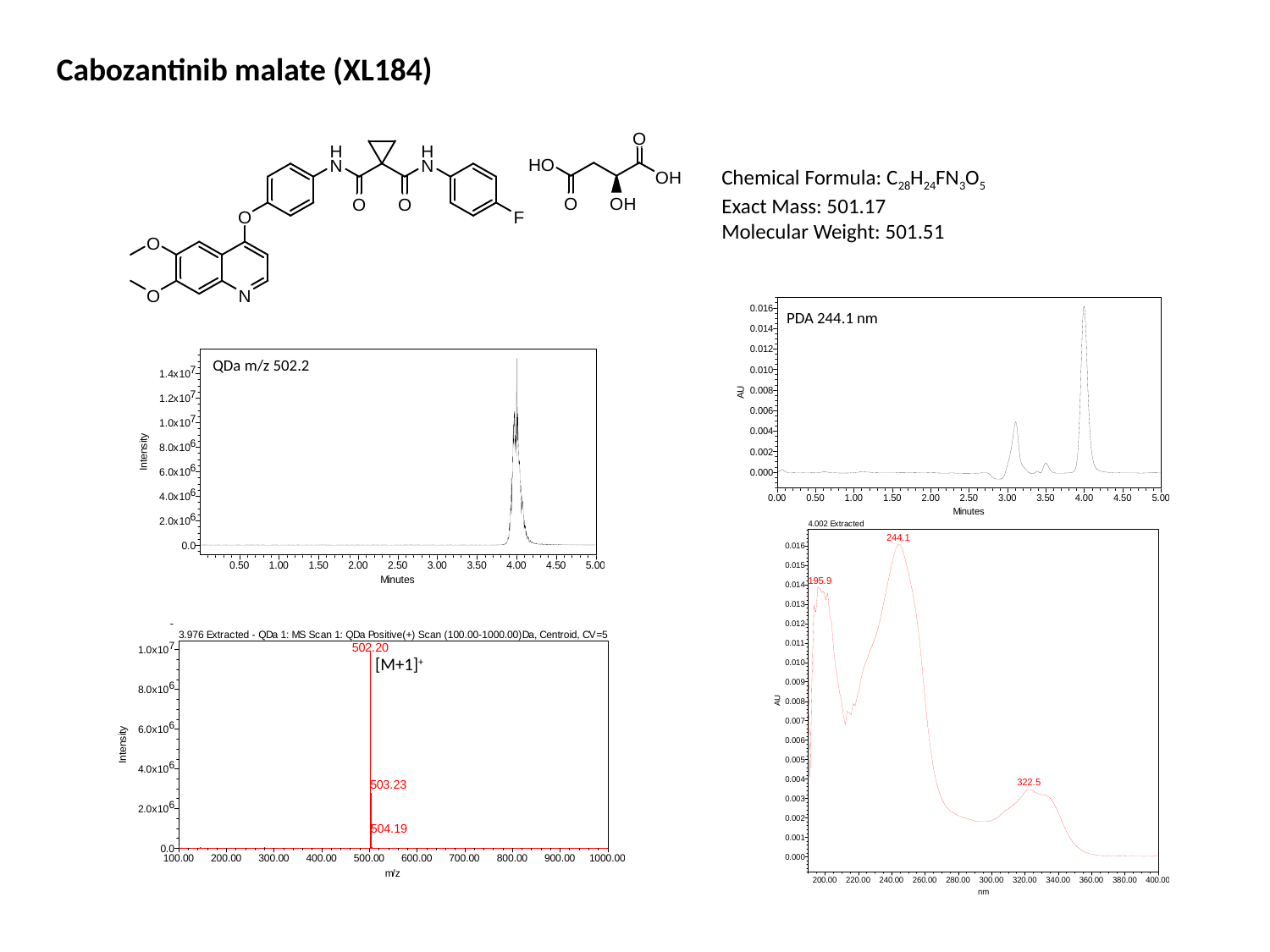

Cabozantinib malate (XL184)
Chemical Formula: C28H24FN3O5
Exact Mass: 501.17
Molecular Weight: 501.51
PDA 244.1 nm
QDa m/z 502.2
[M+1]+

## Slide 18
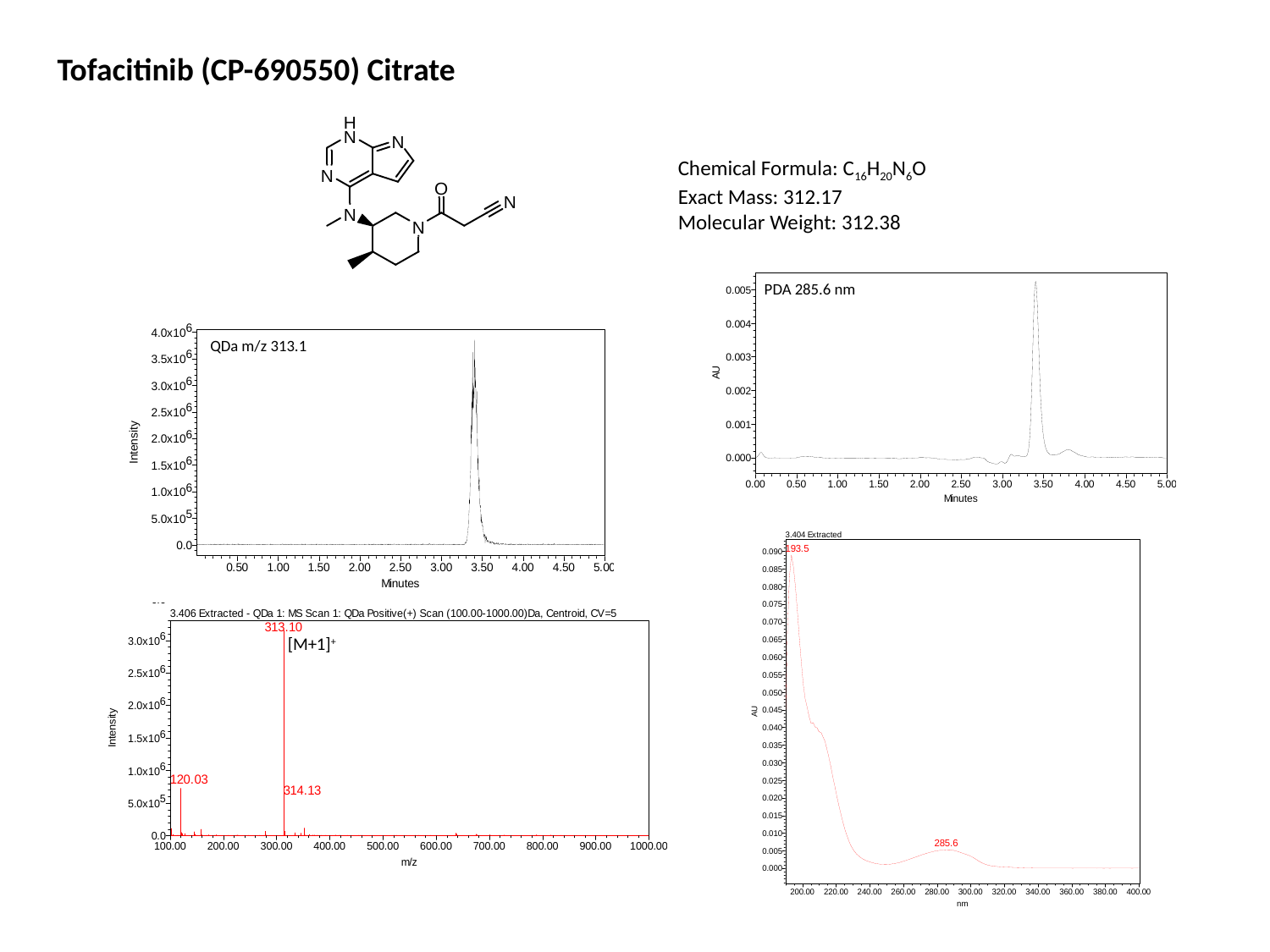

Tofacitinib (CP-690550) Citrate
Chemical Formula: C16H20N6O
Exact Mass: 312.17
Molecular Weight: 312.38
PDA 285.6 nm
QDa m/z 313.1
[M+1]+

## Slide 19
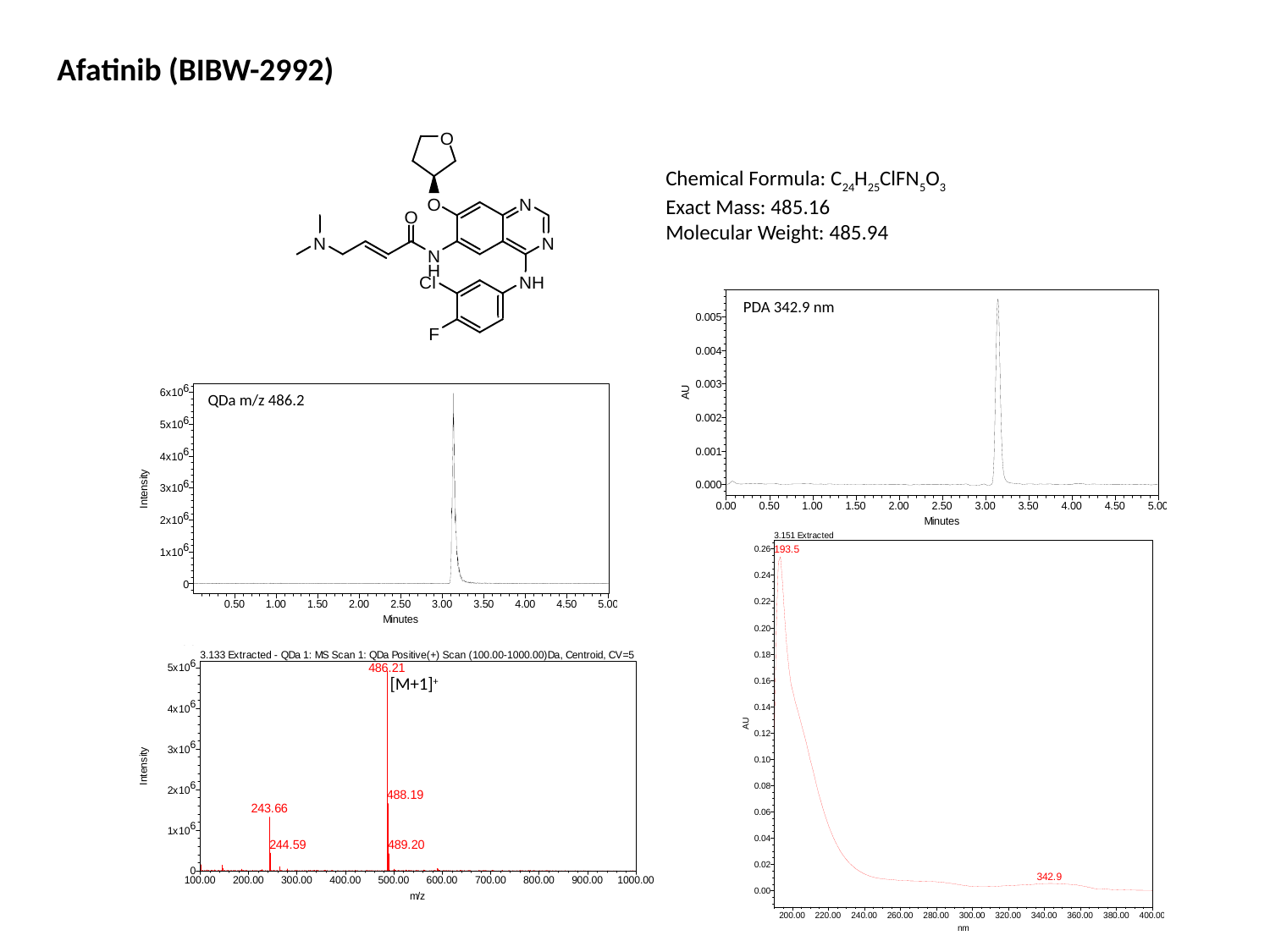

Afatinib (BIBW-2992)
Chemical Formula: C24H25ClFN5O3
Exact Mass: 485.16
Molecular Weight: 485.94
PDA 342.9 nm
QDa m/z 486.2
[M+1]+

## Slide 20
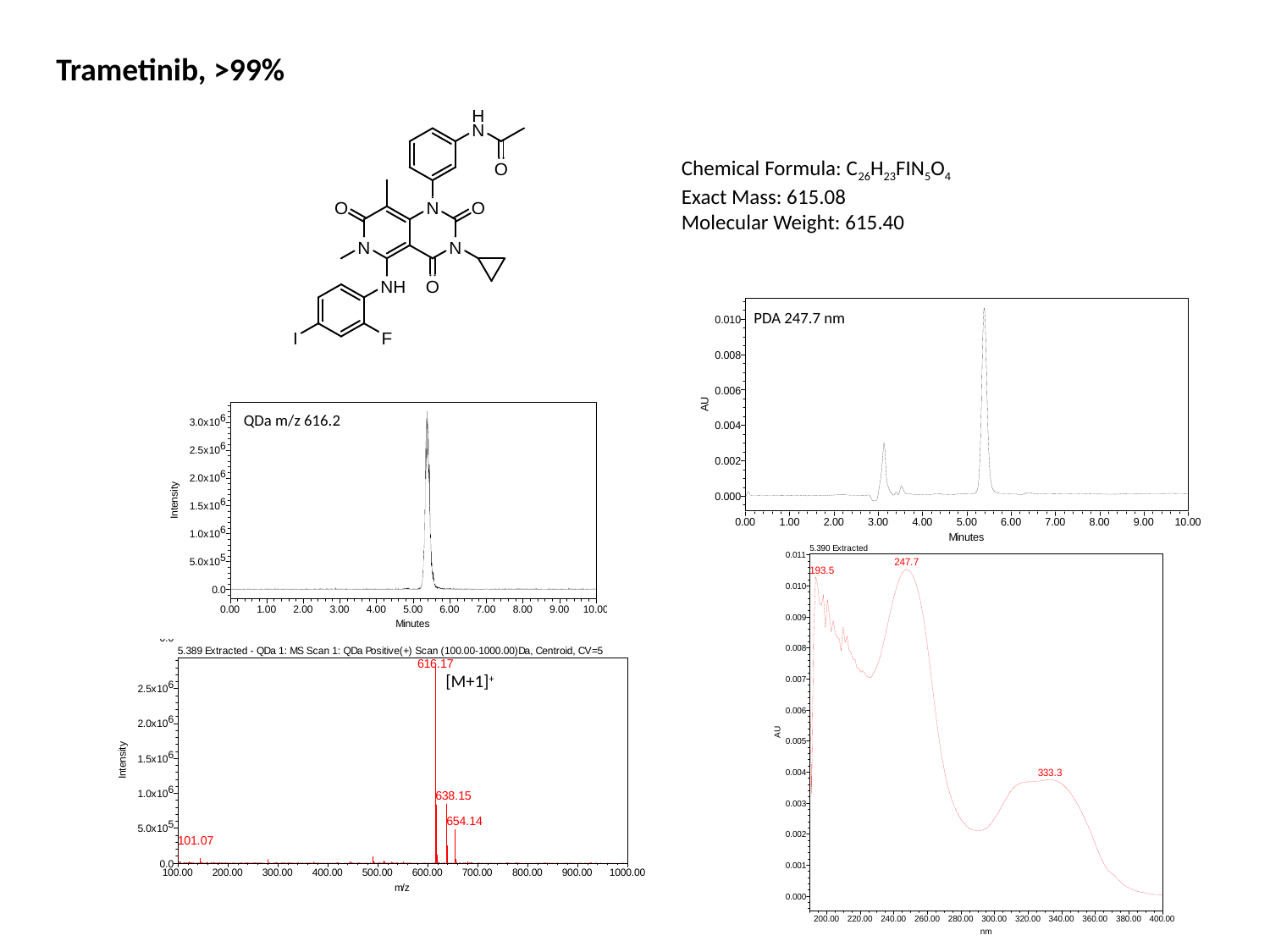

Trametinib, >99%
Chemical Formula: C26H23FIN5O4
Exact Mass: 615.08
Molecular Weight: 615.40
PDA 247.7 nm
QDa m/z 616.2
[M+1]+

## Slide 21
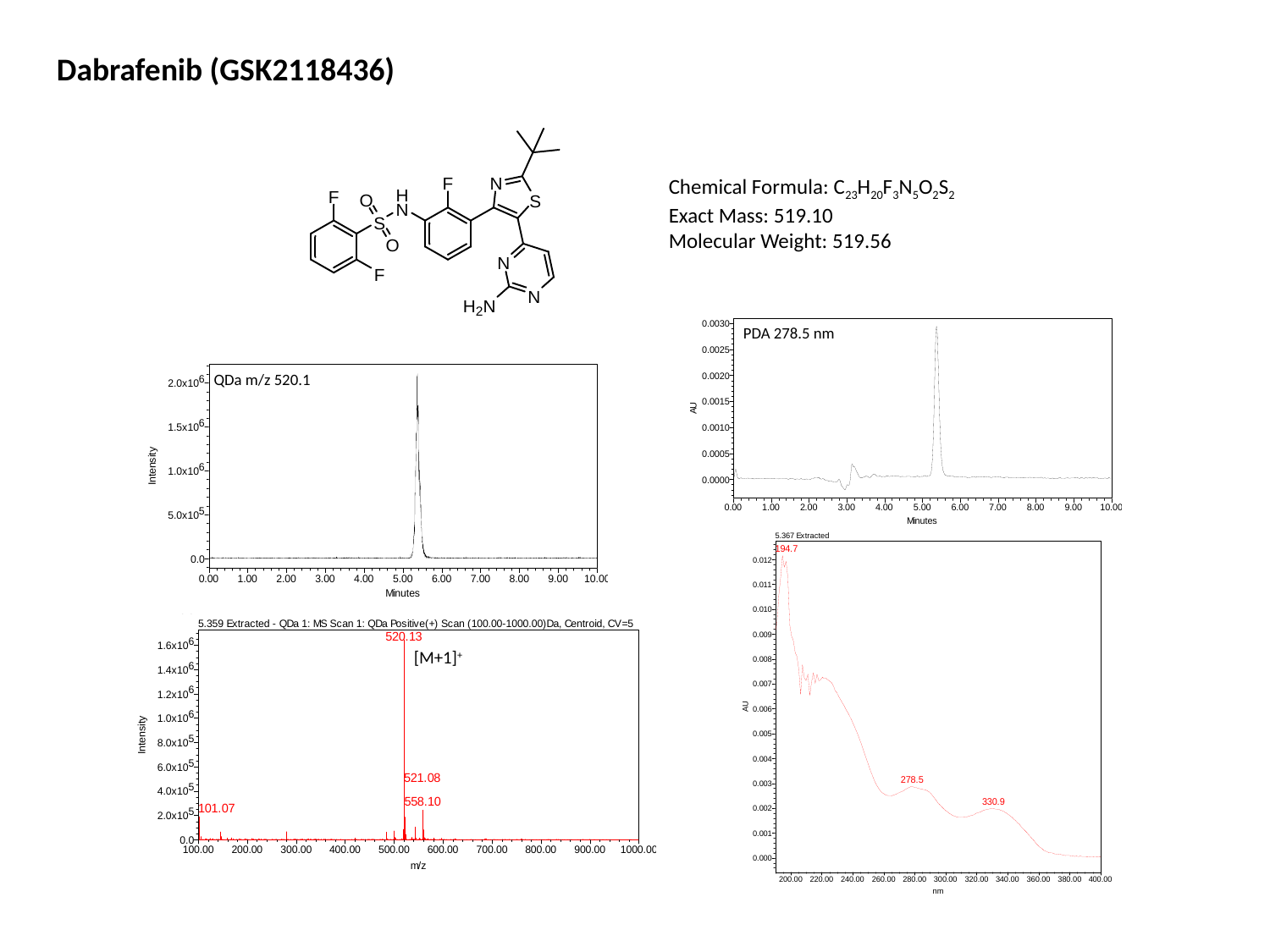

Dabrafenib (GSK2118436)
Chemical Formula: C23H20F3N5O2S2
Exact Mass: 519.10
Molecular Weight: 519.56
PDA 278.5 nm
QDa m/z 520.1
[M+1]+

## Slide 22
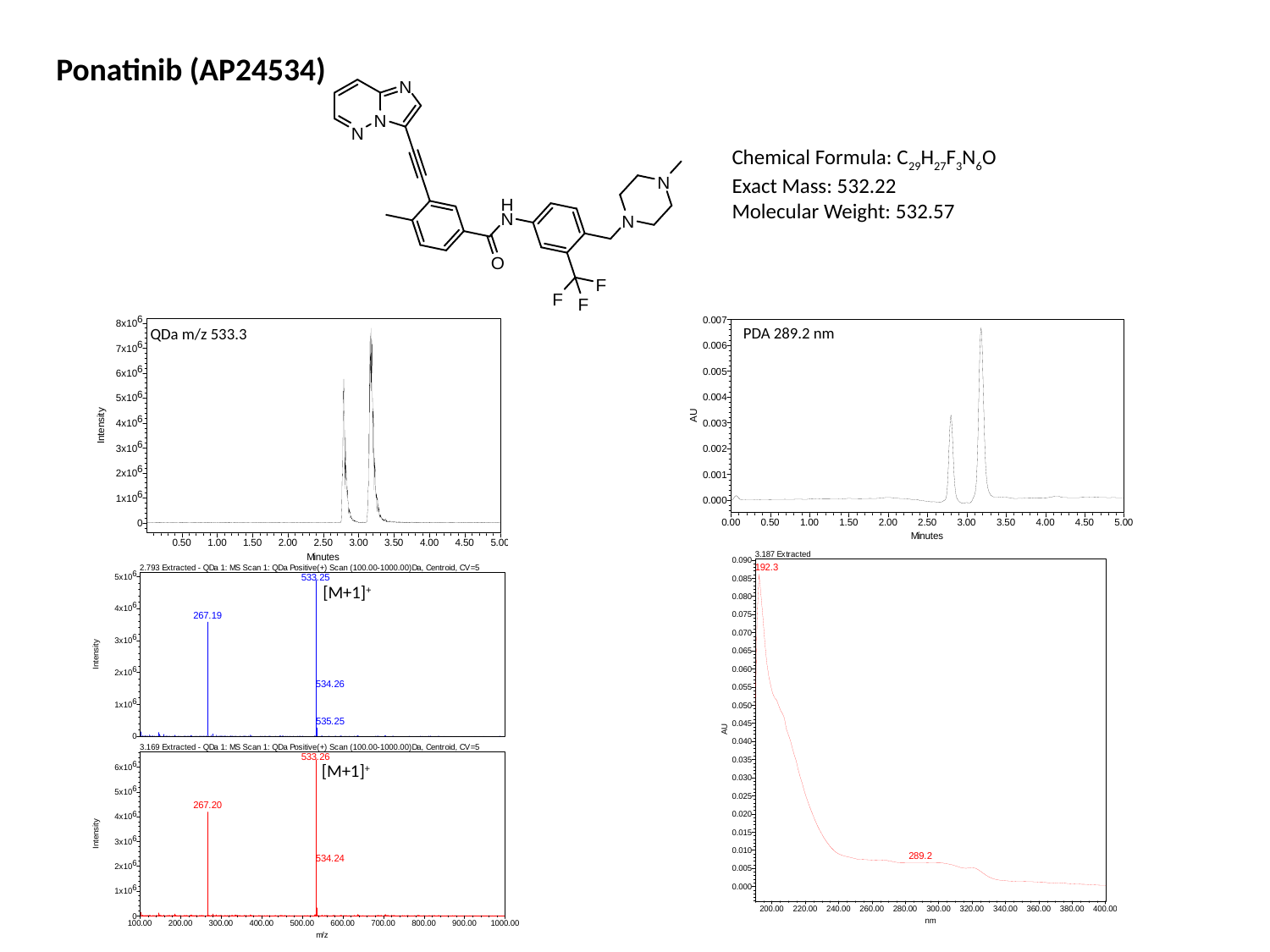

Ponatinib (AP24534)
Chemical Formula: C29H27F3N6O
Exact Mass: 532.22
Molecular Weight: 532.57
PDA 289.2 nm
QDa m/z 533.3
[M+1]+
[M+1]+

## Slide 23
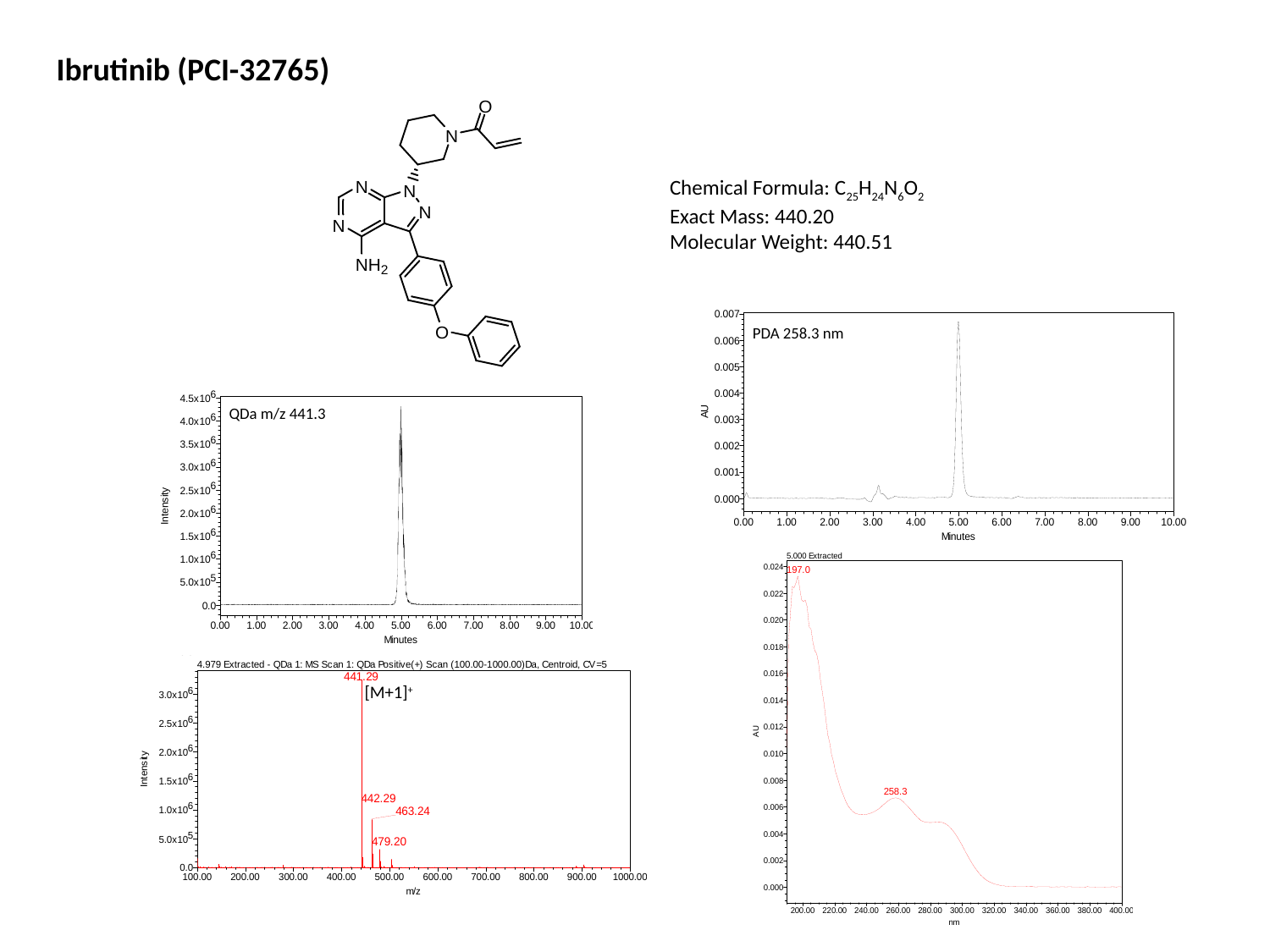

Ibrutinib (PCI-32765)
Chemical Formula: C25H24N6O2
Exact Mass: 440.20
Molecular Weight: 440.51
PDA 258.3 nm
QDa m/z 441.3
[M+1]+

## Slide 24
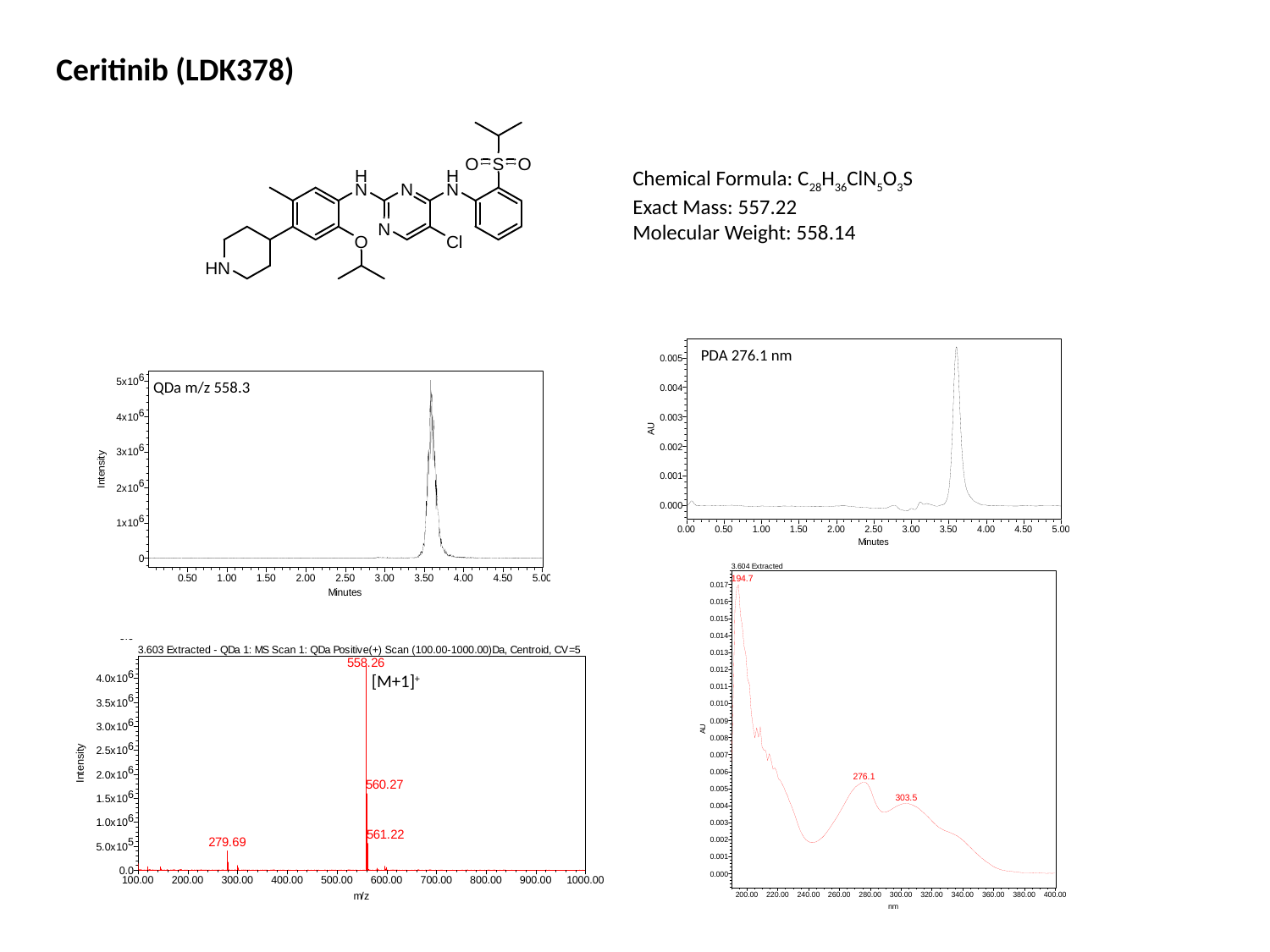

Ceritinib (LDK378)
Chemical Formula: C28H36ClN5O3S
Exact Mass: 557.22
Molecular Weight: 558.14
PDA 276.1 nm
QDa m/z 558.3
[M+1]+

## Slide 25
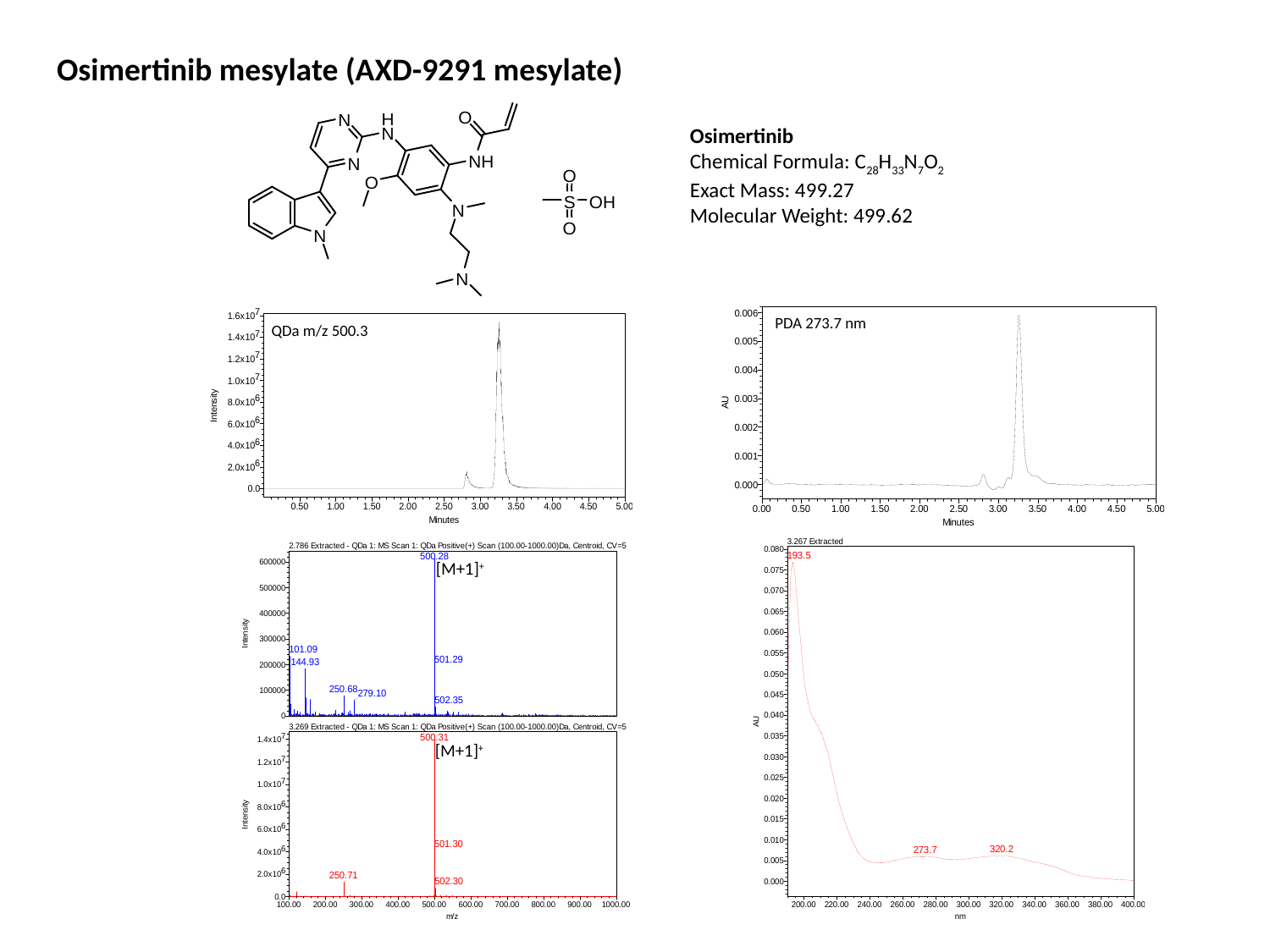

Osimertinib mesylate (AXD-9291 mesylate)
Osimertinib
Chemical Formula: C28H33N7O2
Exact Mass: 499.27
Molecular Weight: 499.62
PDA 273.7 nm
QDa m/z 500.3
[M+1]+
[M+1]+

## Slide 26
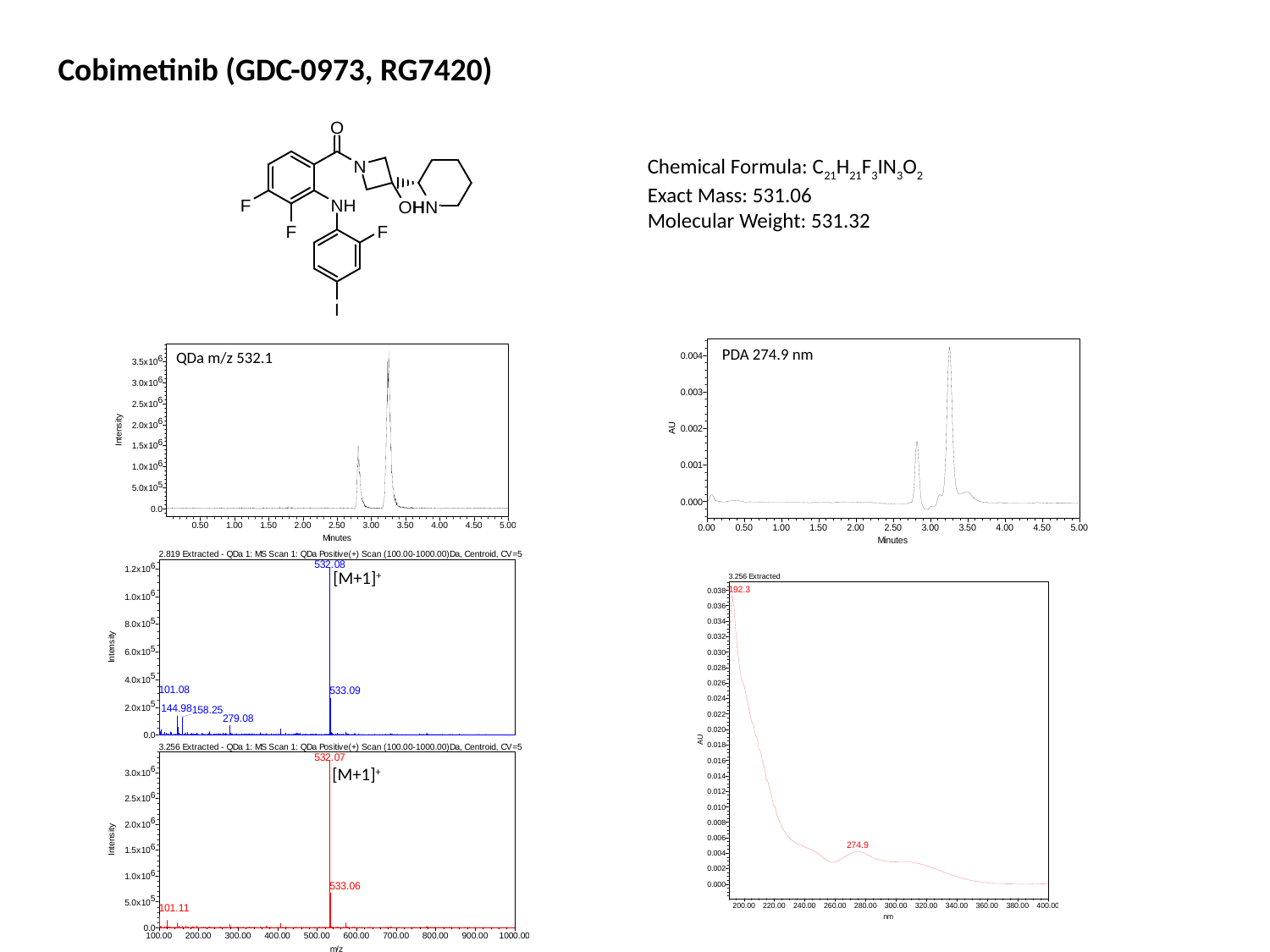

Cobimetinib (GDC-0973, RG7420)
Chemical Formula: C21H21F3IN3O2
Exact Mass: 531.06
Molecular Weight: 531.32
PDA 274.9 nm
QDa m/z 532.1
[M+1]+
[M+1]+

## Slide 27
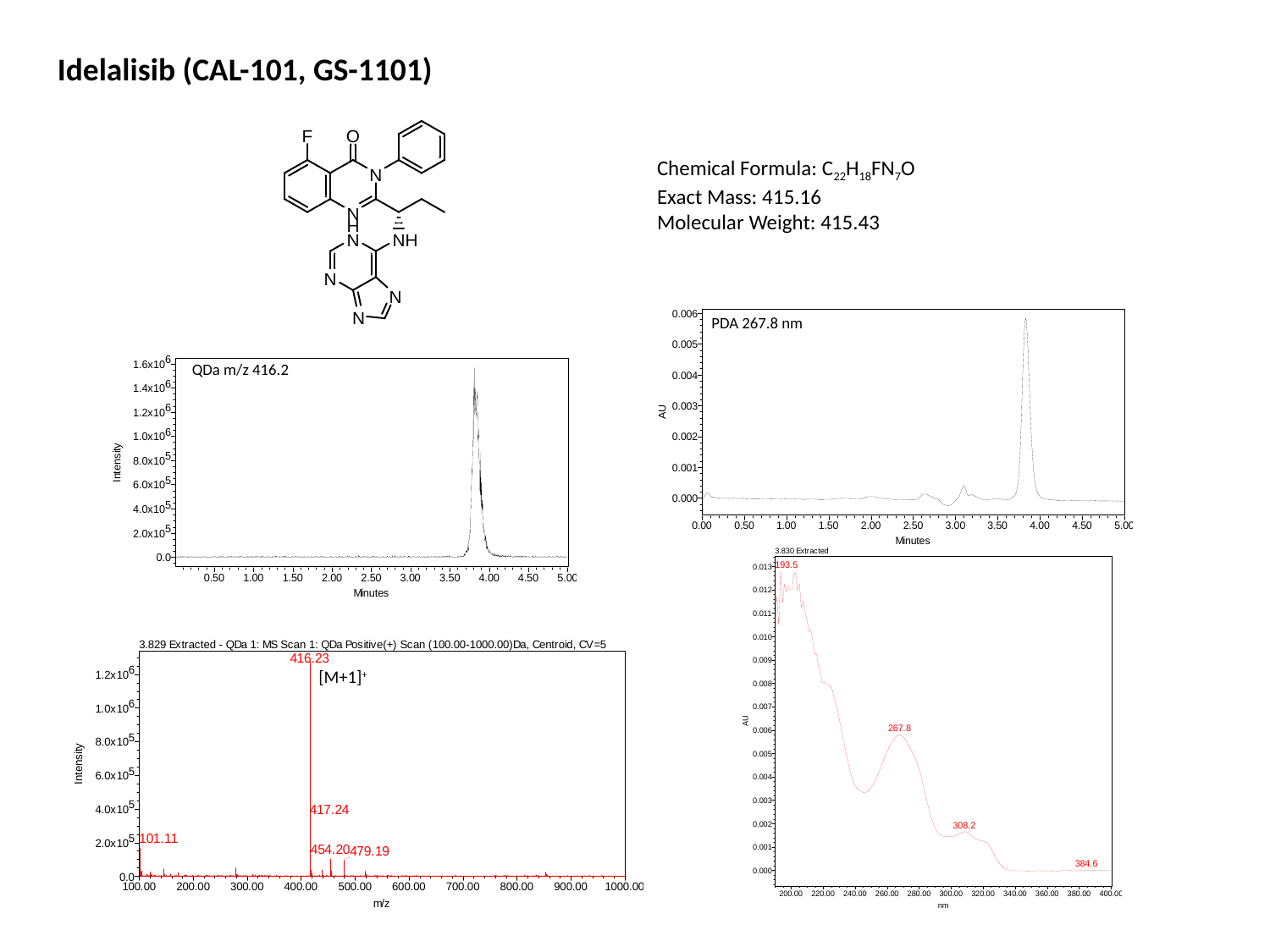

Idelalisib (CAL-101, GS-1101)
Chemical Formula: C22H18FN7O
Exact Mass: 415.16
Molecular Weight: 415.43
PDA 267.8 nm
QDa m/z 416.2
[M+1]+

## Slide 28
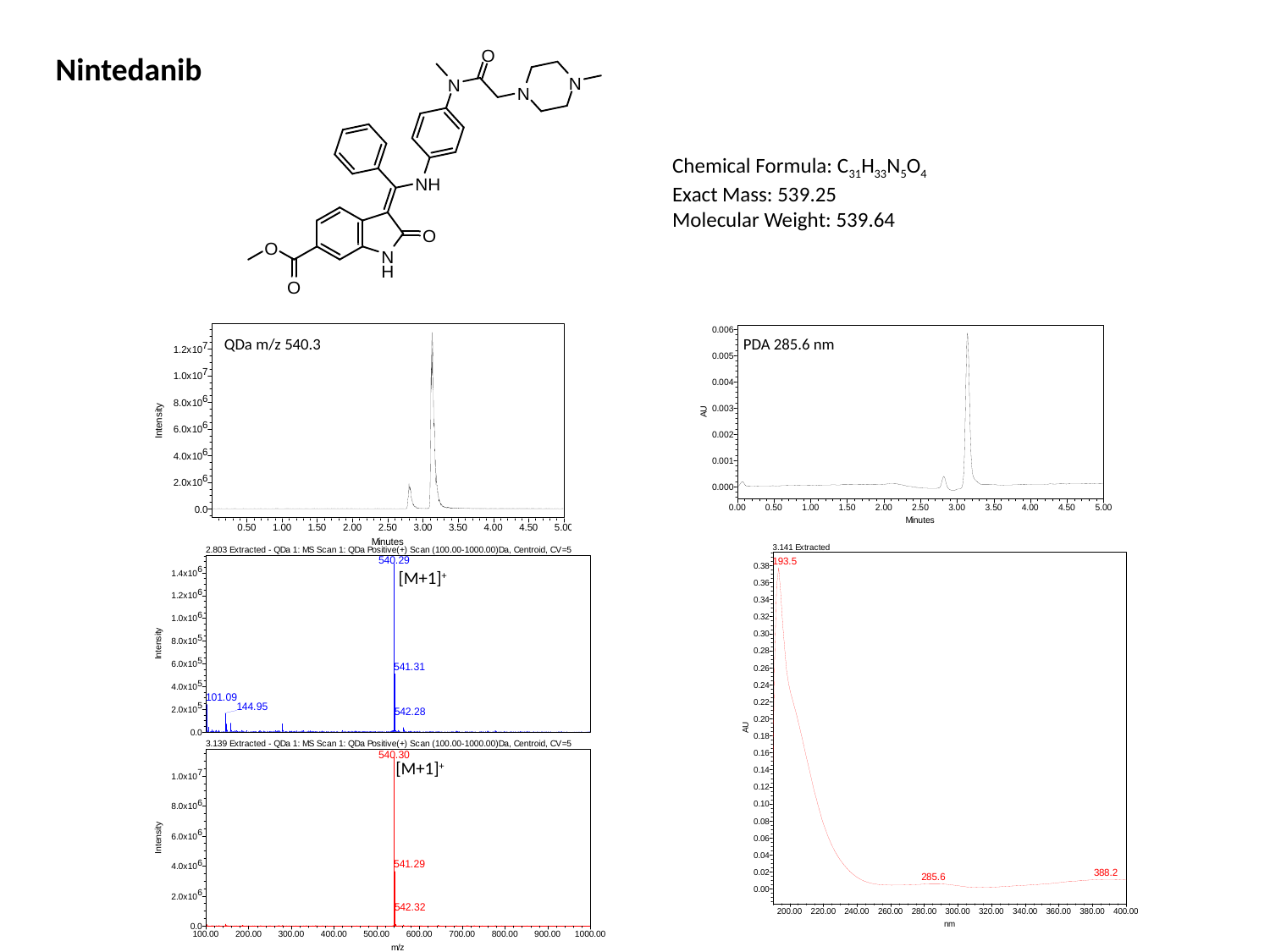

Nintedanib
Chemical Formula: C31H33N5O4
Exact Mass: 539.25
Molecular Weight: 539.64
QDa m/z 540.3
PDA 285.6 nm
[M+1]+
[M+1]+

## Slide 29
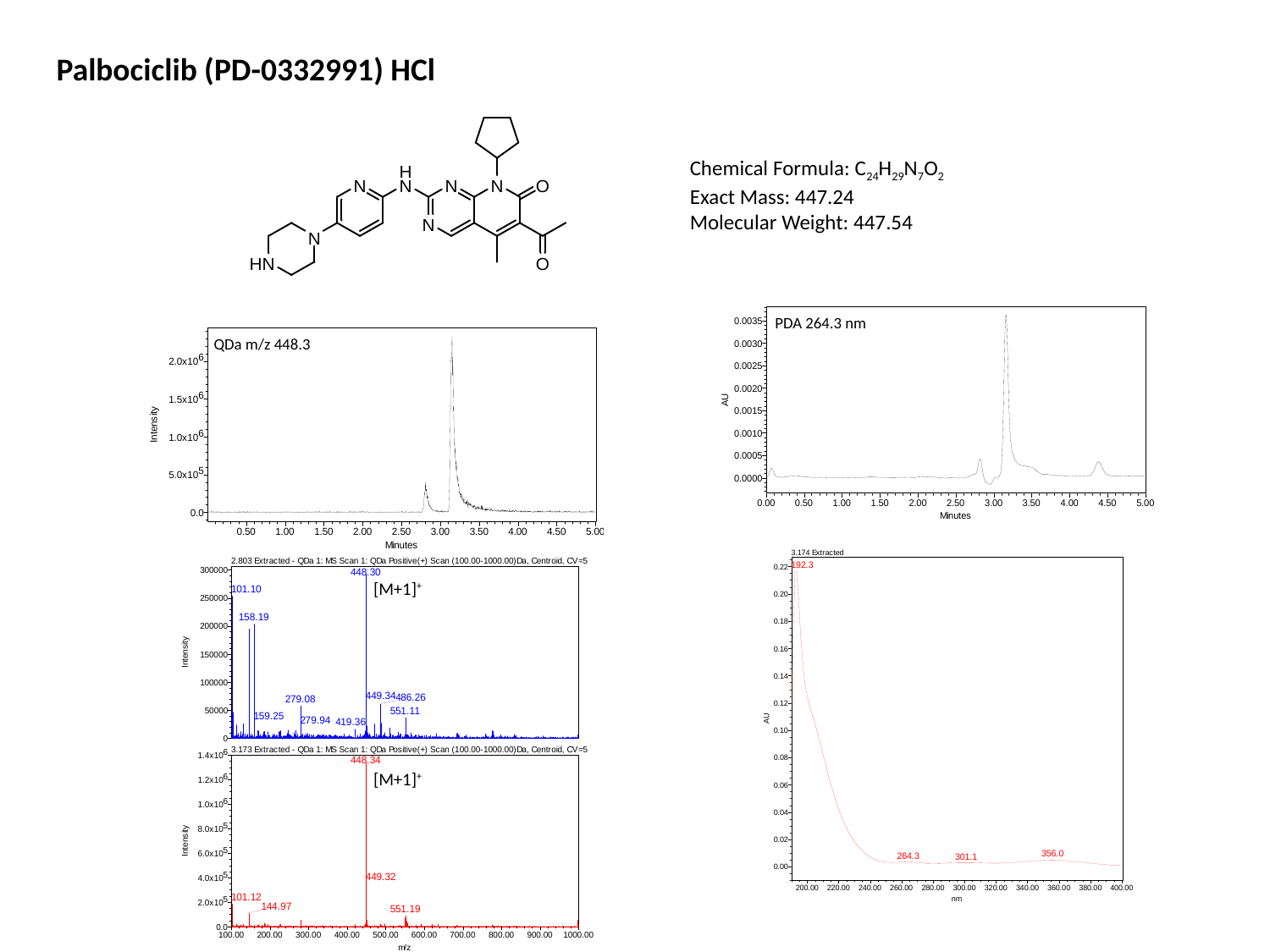

Palbociclib (PD-0332991) HCl
Chemical Formula: C24H29N7O2
Exact Mass: 447.24
Molecular Weight: 447.54
PDA 264.3 nm
QDa m/z 448.3
[M+1]+
[M+1]+

## Slide 30
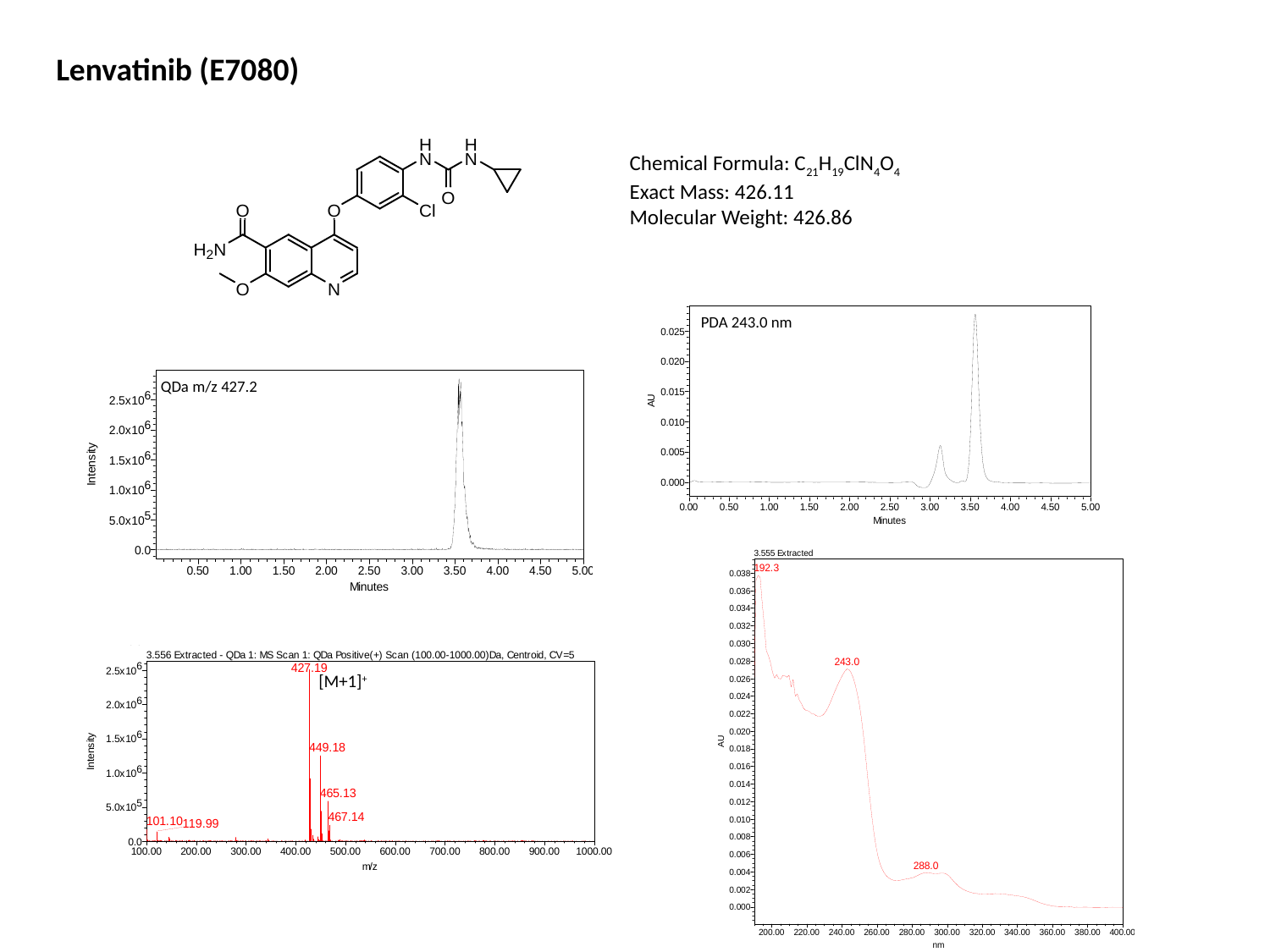

Lenvatinib (E7080)
Chemical Formula: C21H19ClN4O4
Exact Mass: 426.11
Molecular Weight: 426.86
PDA 243.0 nm
QDa m/z 427.2
[M+1]+

## Slide 31
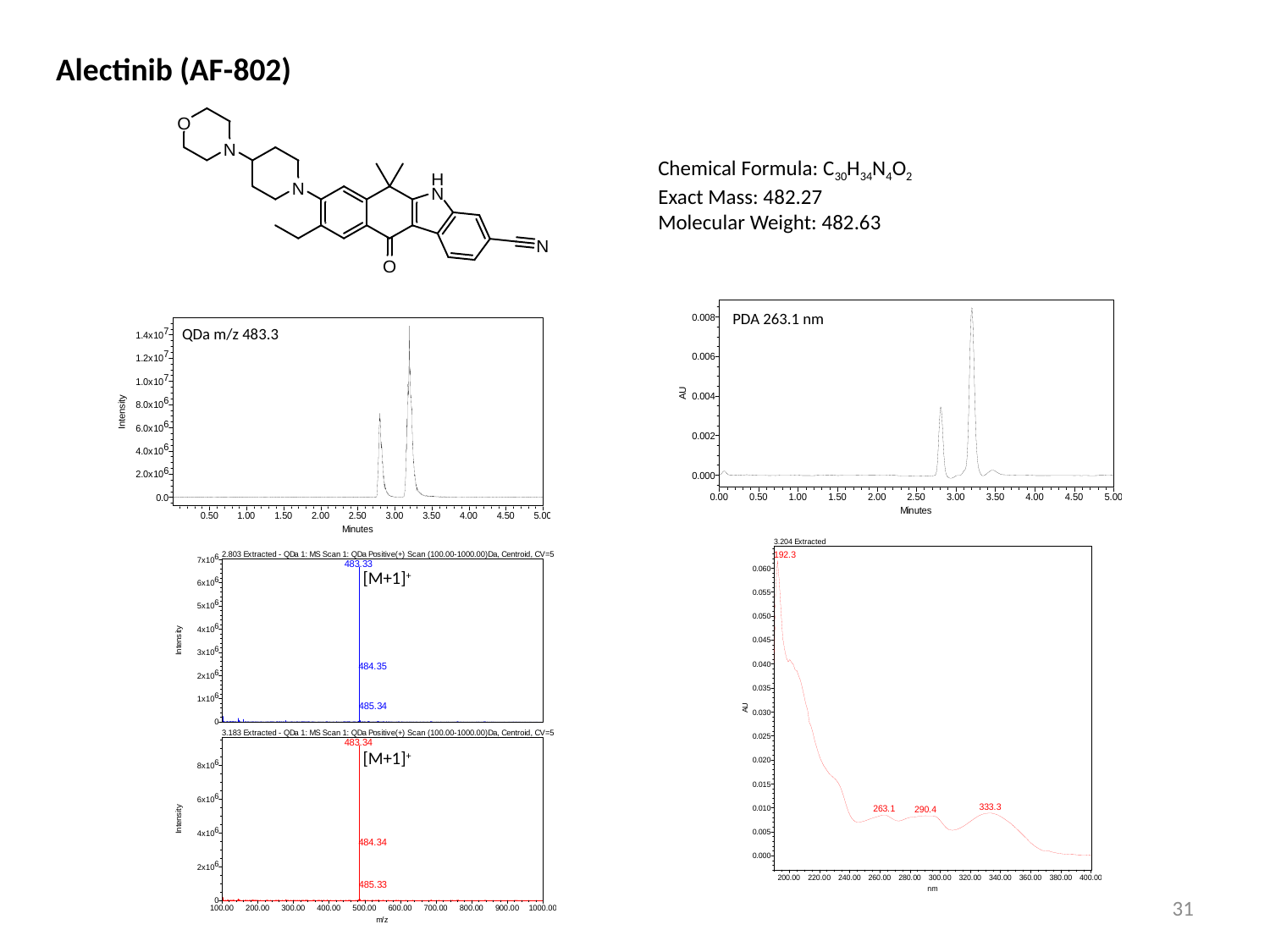

Alectinib (AF-802)
Chemical Formula: C30H34N4O2
Exact Mass: 482.27
Molecular Weight: 482.63
PDA 263.1 nm
QDa m/z 483.3
[M+1]+
[M+1]+
31
