# Supplementary figures and images for: Effects of 31 FDA approved small-molecule kinase inhibitors on isolated rat liver mitochondria
Source: Arch Toxicol. 2016 Dec 28;91(8):2921–38. doi: 10.1007/s00204-016-1918-1 (PMC5515969; doi:10.1007/s00204-016-1918-1)

## Slide 1
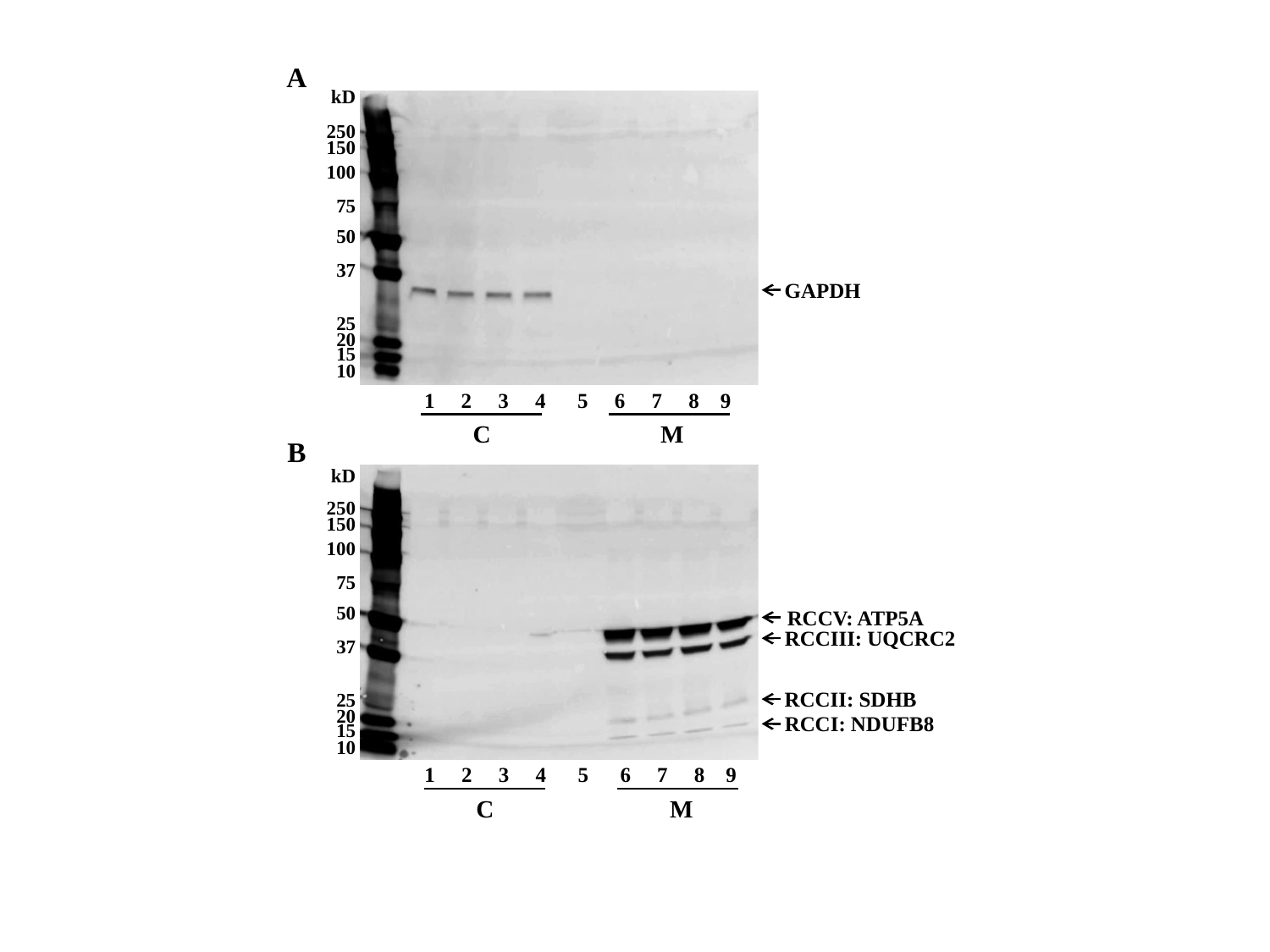

A
kD
250
150
100
75
50
37
GAPDH
25
20
15
10
1 2 3 4 5 6 7 8 9
C
M
B
kD
250
150
100
75
50
RCCV: ATP5A
RCCIII: UQCRC2
37
RCCII: SDHB
25
20
RCCI: NDUFB8
15
10
1 2 3 4 5 6 7 8 9
C
M

Supplement: Supplementary file 2 — Supplementary material 2 (PPTX 115 kb) [file 204_2016_1918_MOESM2_ESM.pptx]
